# Macrophage membrane functionalized biomimetic nanoparticles for targeted anti-atherosclerosis applications

**DOI:** 10.7150/thno.47841

**Published:** 2021-01-01

**Authors:** Yi Wang, Kang Zhang, Tianhan Li, Ali Maruf, Xian Qin, Li Luo, Yuan Zhong, Juhui Qiu, Sean McGinty, Giuseppe Pontrelli, Xiaoling Liao, Wei Wu, Guixue Wang

**Affiliations:** 1 Key Laboratory for Biorheological Science and Technology of Ministry of Education, State and Local Joint Engineering Laboratory for Vascular Implants, Bioengineering College of Chongqing University, Chongqing, 400030, China.; 2 Chongqing Key Laboratory of Nano/Micro Composite Material and Device, School of Metallurgy and Materials Engineering, Chongqing University of Science and Technology, Chongqing, 401331, China.; 3 Division of Biomedical Engineering, University of Glasgow, UK.; 4 Istituto per le Applicazioni del Calcolo - CNR, Via dei Taurini 19, 00185, Roma, Italy.

**Keywords:** macrophage membrane, biomimetic, targeted delivery, atherosclerosis, ApoE knockout mice

## Abstract

Atherosclerosis (AS), the underlying cause of most cardiovascular events, is one of the most common causes of human morbidity and mortality worldwide due to the lack of an efficient strategy for targeted therapy. In this work, we aimed to develop an ideal biomimetic nanoparticle for targeted AS therapy.

**Methods:** Based on macrophage “homing” into atherosclerotic lesions and cell membrane coating nanotechnology, biomimetic nanoparticles (MM/RAPNPs) were fabricated with a macrophage membrane (MM) coating on the surface of rapamycin-loaded poly (lactic-co-glycolic acid) copolymer (PLGA) nanoparticles (RAPNPs). Subsequently, the physical properties of the MM/RAPNPs were characterized. The biocompatibility and biological functions of MM/RAPNPs were determined *in vitro*. Finally, in AS mouse models, the targeting characteristics, therapeutic efficacy and safety of the MM/RAPNPs were examined.

**Results:** The advanced MM/RAPNPs demonstrated good biocompatibility. Due to the MM coating, the nanoparticles effectively inhibited the phagocytosis by macrophages and targeted activated endothelial cells *in vitro*. In addition, MM-coated nanoparticles effectively targeted and accumulated in atherosclerotic lesions *in vivo*. After a 4-week treatment program, MM/RAPNPs were shown to significantly delay the progression of AS. Furthermore, MM/RAPNPs displayed favorable safety performance after long-term administration.

**Conclusion:** These results demonstrate that MM/RAPNPs could efficiently and safely inhibit the progression of AS. These biomimetic nanoparticles may be potential drug delivery systems for safe and effective anti-AS applications.

## Introduction

Atherosclerosis (AS) is a typical chronic inflammatory vascular disease characterized by the gradual thickening of arterial walls 1,2. It is the predominant pathological onset of cardiovascular diseases (CVDs), the main cause of death in many parts of the world 3,4. Strong evidence has indicated that oral statins reduce the risk of atherosclerotic CVD for primary and secondary prevention 5. However, these oral drug therapies suffer from a number of issues, including poor bioavailability, slow therapeutic efficacy, and serious side effects. Targeted drug delivery by nanotechnology has been successfully used for the systemic delivery of a variety of drug molecules, in many cases demonstrating an enhancement in therapeutic efficacy and mitigation of side effects compared to freely administered drugs 6-8. Recent advances have shown the potential of nanomedicine-based treatment strategies for cardiovascular diseases 9-12. However, like any strategy, there are limitations on the use of targeted drug delivery by nanoparticles (NPs). Clearance by the immune system before a nanoparticle can reach its target is one of the major hurdles that almost all platforms must overcome 13-15.

In recent years, cell membrane coating nanotechnology has emerged as a promising therapeutic platform 16-18. By fusing natural cell membranes onto synthetic NPs, these NPs inherit the specific biological functions of the source cells, such as long circulation and disease-relevant targeting 19,20. For instance, Tasciotti et al. reported the first leukocyte membrane-coated nanoparticles that enhanced circulation time and improved tumoritropic accumulation 21. Zhang et al. recently reported neutrophil membrane-coated nanoparticles to alleviate inflammatory arthritis and platelet membrane-coated metal-organic framework nanoparticles to target gene silencing *in vivo* 22,23. Most recently, cell membrane coating nanotechnology has been applied to treat certain cardiovascular diseases 24. For instance, it has been reported that platelet membrane-coated NPs have been applied to detect and treat atherosclerosis 25,26. In our previous study, red blood cell (RBC)-coated nanoparticles enabled the safe and efficient management of atherosclerosis 27. These works provide a promising platform and lay the foundation for exploiting more sophisticated cell membrane-based nanotherapeutics against atherosclerosis.

Macrophages are large and highly versatile white blood cells that intrinsically work as major cellular effectors in inflammatory and tissue repair processes 28,29. In previous studies, macrophage membrane-coated NPs have demonstrated high targeted delivery efficiency to various inflammatory diseases as well as decent therapeutic efficacy, including rheumatoid arthritis, cancer and sepsis 30-34. Many studies have shown that macrophages also play a major role in the pathogenesis of AS 35-37. During the early stage of AS, macrophage colony-stimulating factors and other differentiation factors drive monocytes to differentiate into macrophages. During the development of AS, macrophages promote plaque formation 38. In fact, surface proteins on the macrophage membrane play a dominant role in AS pathology 39. In particular, integrin α4β1, for its “homing” into atherosclerotic lesions, can actively bind to vascular cell adhesion molecule-1 (VCAM-1), which is highly expressed in the inflamed endothelium 30,40. In addition, a recent study reported that macrophage membrane-coated nanoparticles have the ability to target AS 41. This evidence indicates that macrophages have an inherent affinity for atherosclerotic lesions, suggesting that a macrophage membrane-coated drug delivery system may be a powerful platform for the targeted treatment of AS.

**Figure 1:**
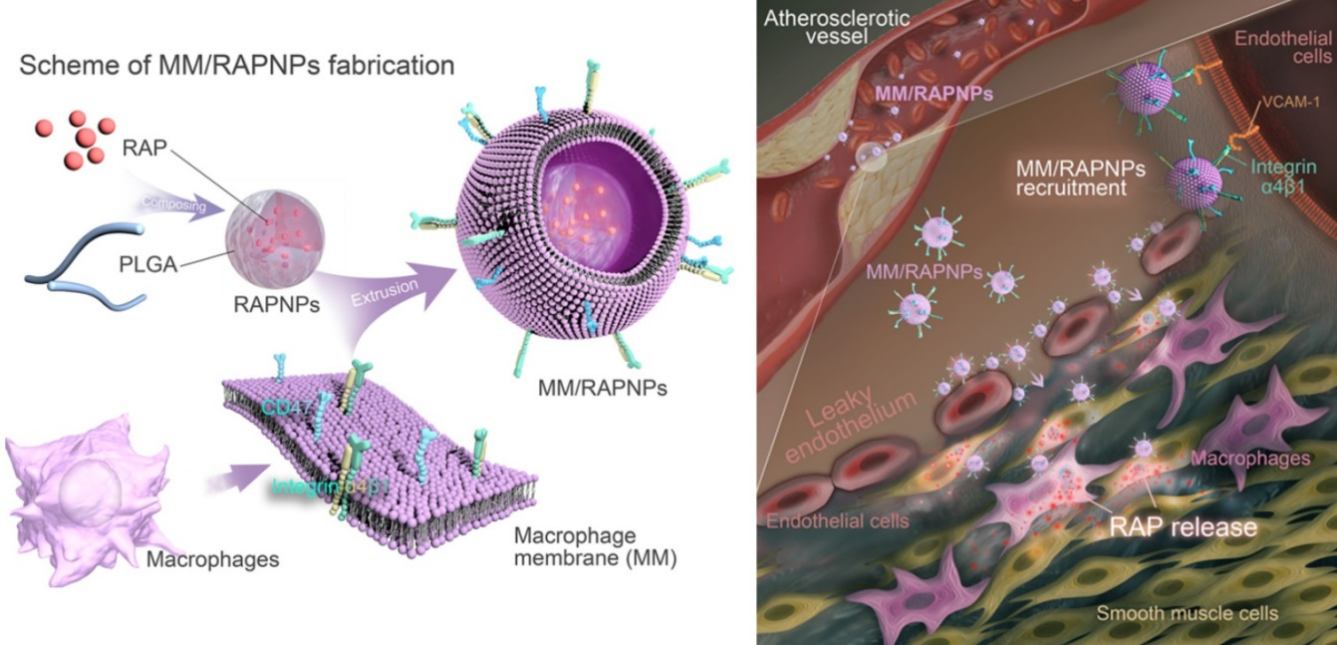
Schematic of MM/RAPNP fabrication and its treatment for AS.

Therefore, in this study, we sought to construct macrophage membrane (MM)-coated biomimetic nanoparticles for the targeted therapy of AS. Rapamycin (RAP) is an inhibitor of the mammalian target of RAP (mTOR) pathway, which exhibits multiple pharmacological functions, including anti-inflammatory and anti-proliferative activities and autophagy activation 42. Various RAP-based agents, including oral drugs and nanomedicines, have been widely used to manage atherosclerosis 43-45. Therefore, RAP was used as a model drug in this work. Specifically, we camouflaged poly(lactic-co-glycolic acid) (PLGA) NPs loaded with RAP (RAPNPs) with MMs for the targeted and efficient management of atherosclerosis (Figure 1). We hypothesized that the resulting MM-coated RAPNPs (MM/RAPNPs) could be targeted towards and accumulate within atherosclerotic plaques to locally release antiatherosclerotic drugs, thereby inhibiting the progression of AS.

## Methods

### Materials

RAP and PLGA (MW 90000, 50:50) were purchased from Dalian Meilun Biotechnology Co., Ltd. (Dalian, Chia). 1,19-Dioctadecyl-3,3,39,39-tetramethylindodicarbocyanine perchlorate (DiD) was purchased from Biotium Inc. (Fremont, US). DiO, DAPI, Cell Total Protein Extraction kits and Membrane Protein Extraction kits were supplied by Beyotime Institute of Biotechnology (Jiangsu, China). The CellTiter 96^TM^ AQueous One Solution Cell Proliferation Assay (MTS) was purchased from Thermo Fisher Scientific (SanJose, CA, USA). Lipopolysaccharide (LPS) was purchased from Solarbio (Beijing, China). The mouse glycoprotein ELISA kit, mouse TNF-α ELISA kit and mouse IL-6 ELISA kit were purchased from Wuhan Colorful Gene Biological Technology Co., Ltd. (Wuhan, China). LysoTracker Green was purchased from Yeasen Biotech Co. Ltd. (Shanghai China). Ultrapure water with a resistivity of 18.2 MΩ·cm was used throughout the experiments.

### Preparation of the macrophage membranes

Macrophage membranes were isolated from RAW264.7 cells as previously described, with a minor modification 30,46. The RAW264.7 cell membrane was obtained using the Membrane Protein Extraction kit. Briefly, collected cells were dispersed in membrane protein extraction buffer solutions and cooled in an ice bath for 15 min. After that, the cell suspension was transferred to a glass homogenizer and homogenized approximately 30 times. Then, the obtained mixture was centrifuged (1500 rpm, 10 min, 4 °C and 14000 rpm, 30 min) to acquire the cell membranes. A bicinchoninic acid (BCA) protein assay was employed to analyze the total protein content in the obtained macrophage membrane. In order to obtain MM vesicles, the extracted macrophage membranes were first ultrasonicated for 15 min and then extruded 10 times through a 400 nm polycarbonate porous membrane using an Avestin mini extruder (Avestin, LF-1, Canada). The harvested MM vesicles were stored in water at 4 °C.

### Preparation of rapamycin-loaded PLGA nanoparticles (RAPNPs)

RAPNPs were prepared *via* the nanoprecipitation method as previously described, with a slight modification 47,48. Briefly, RAP (1.5 mg) and PLGA (15 mg) were dissolved in DMSO (1 mL). The mixture was precipitated by adding 4 mL of water dropwise with gentle stirring, and then the mixture was transferred to dialyzation (molecular weight cut-off (MWCO) of 3500 Da) against water to remove free RAP and DMSO. The RAPNP solution was quantified and stored at 4 °C. To prepare the fluorescently labeled nanoparticles, 0.1 wt% DiD (excitation = 644 nm, emission= 665 nm) was loaded into PLGA according to the former method (DiDNPs).

### Preparation of MM camouflaged RAPNPs (MM/RAPNPs)

MM/RAPNPs were fabricated by coating RAPNPs with MMs by a direct extrusion method. Briefly, MM vesicles and RAPNPs were mixed at a membrane protein-to-polymer ratio of 1:1 (w/w) and sonicated for 3 min in a sonicator bath (FS30D, 42 kHz, 100 W). The mixture was then extruded 10 times through a 200 nm polycarbonate porous membrane using an Avestin mini extruder (Avestin, LF-1, Canada) to harvest the MM/RAPNPs.

### Characterization of the nanoparticles

The size, size distribution and zeta potentials of RAPNPs, MM vesicles and MM/RAPNPs were determined using a Malvern Zetasizer Nano ZS unit (Nano ZS 90, Malvern, U.K.) with a He-Ne laser (λ = 633 nm) at a scattering angle of 90° at 25 °C. A drop of NP solution at a concentration of 100 μg/mL was deposited onto a glow-discharged carbon-coated grid and stained with 1% phosphotungstic acid. Subsequently, the morphologies of the RAPNPs and MM/RAPNPs were visually observed using transmission electron microscopy (TEM) at 200 kV (JEM-2100F, JEOL, Japan).

### Identification of the membrane orientation of MM/RAPNPs

The membrane orientation of MM/RAPNPs was identified by quantifying the glycoprotein content in the MM/RAPNPs as previously reported 49. Briefly, the MMs extracted from 1×10^7^ cells and the subsequent MM/RAPNPs were incubated with trypsin at room temperature for 2 h to initiate trypsinization. Then, the samples were centrifuged at 8000 rpm for 5 min, and the supernatant was collected to quantify the glycoprotein content using a Mouse Glycoprotein ELISA Kit following the manufacturer's instructions.

### Characterization of proteins

The membrane proteins were characterized by polyacrylamide gel electrophoresis (SDS-PAGE). The membrane proteins of the MM vesicles and MM/RAPNPs were extracted by Cell Total Protein Extraction kits. The extracted membrane proteins were run on a 4-12% Bis-Tris 10-well minigel in running buffer using a Bio-Rad electrophoresis system at 75 V for 0.5 h and then at 140 V for 1 h. Finally, the resulting polyacrylamide gel was stained with SimplyBlue overnight for visualization.

Furthermore, the integrin α4β1 and CD47 contents in RAW264.7 cells, MMs, MM/RAPNPs and RAPNPs were determined by western blot analysis. The total protein of the lysis solution from 1 × 10^7^ RAW264.7 cells, RAPNPs, MMs extracted from 1 × 10^7^ cells and the subsequent MM/RAPNPs were extracted by Cell Total Protein Extraction kits and used for measurements. Samples underwent electrophoresis on a 10% SDS-polyacrylamide gel and were transferred to a polyvinylidene difluoride membrane (Millipore, USA). Then, the membranes were treated with primary antibodies against α4 (anti-integrin α4, 8440S, CST), β1 (anti-integrin β1, 34971, CST), and CD47 (anti-CD47 antibody, ab175388, Abcam), followed by horseradish peroxidase-labeled goat/anti-rabbit IgG (H+L) (Beyotime, Jiangsu, China). The protein signals were measured by the enhanced chemiluminescence method using a ChemiDoc MP imaging system (Bio-Rad, USA).

### Drug loading and *in vitro* drug release study

RAPNPs were first frozen at -80 °C and then freeze-dried with a Labconco Free Zone lyophilizer. Then, the RAPNP lyophilized powder was dissolved in DMSO, and the absorbance was measured with a UV/Vis spectrophotometer (DU730, Beckman Coulter) at 280 nm. According to the preestablished standard curve of RAP in DMSO, the drug loading efficiency (LE) and drug encapsulation efficiency (EE) were calculated as follows:


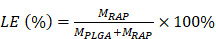
(1)


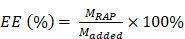
 (2)

where *M*_RAP_ is the mass of RAP loaded in the NPs, *M*_PLGA_ is the mass of polymer in the formulation and *M*_added_ is the mass of RAP added.

The drug (RAP) release from RAPNPs and MM/RAPNPs was studied separately using a dialysis method. Briefly, RAPNP and MM/RAPNP solutions (2 mg/mL, 1 mL each) were added to disposable dialysis bags (MWCO: 3500 Da, Thermo Scientific). The dialysis bags were then immersed in 10 mL of phosphate-buffered saline (PBS) solution (release medium, pH 7.4) at 37 °C. Three independent replicates were used for each sample. One milliliter of release medium was collected for analysis at different time intervals and replaced with an equivalent volume of fresh PBS at 37 °C. The cumulative amount of RAP released was quantified by a UV/Vis spectrophotometer (DU730, Beckman Coulter) at 280 nm.

### Colocalization study

The MMs were stained using DiO. Then, the DiO-labeled MMs were coated onto the DiDNPs by a direct extrusion method as previously described. Four microliters of sample was added to the coverslip for observation by confocal laser scanning microscopy (CLSM). For the colocalization study after cell uptake, human umbilical vein endothelial cells (HUVECs) were maintained in DMEM supplemented with 10% fetal bovine serum (FBS) and cultured at 37 °C with 5% CO_2_. Then, 150 μg of MM/DiDNPs was added to the HUVECs. After incubation for an additional 4 h, the cells were washed with PBS three times, fixed with tissue fixative for 30 min at room temperature, and then the nuclei of the cells were stained with 4′,6-diamidino-2-phenylindole (DAPI). The cells were visualized using CLSM.

### Cellular uptake

*In vitro* cellular uptake was evaluated in human umbilical vein endothelial cells (HUVECs) and RAW264.7 cells. For HUVEC uptake, cells were maintained in 1640 medium supplemented with 10% FBS. Confluent cells were stimulated with 50 ng/mL TNF-α (Gibco) for 24 h to activate HUVECs 26. Then, 100 μg of DiDNPs or MM/DiDNPs were added to nonactivated or activated HUVECs. After incubation for 2 h, the cellular uptake of DiDNPs and MM/DiDNPs was quantified by fluorescence-activated cell sorting (FACS) analysis (BD, USA). Cells were also stained with DAPI for visualization under by confocal laser scanning microscopy (CLSM) (Olympus, Japan). To investigate the importance of VCAM-1 on the interaction and cellular uptake of MM-coated NPs, activated HUVECs were treated with 300 μg/mL VCAM-1 antibodies for 1 h before incubation with MM-coated nanoparticles. Then, after incubation with 100 μg of MM/DiDNPs for 2 h, the cells were stained with DAPI for visualization by CLSM.

Similarly, to evaluate the effects of MM camouflaging on phagocytosis reduction by macrophages, experiments were performed using RAW264.7 macrophage cells. The internalization of DiDNPs and MM/DiDNPs by macrophage cells was evaluated by CLSM and FACS measurements. Briefly, RAW264.7 macrophage cells were seeded in 12-well plates at a density of 1×10^5^ cells per well in 1 mL of DMEM supplemented with 10% FBS and cultured overnight. Then, 150 μg of DiDNPs or MM/DiDNPs were added to each well. After incubation for 0.5, 1, 2, and 4 h, the nuclei were stained with DAPI for CLSM imaging. Cells were collected for quantification by FACS analysis. To understand where the nanoparticles located to in the cytoplasm, lysosomes were stained with 50 nM LysoTracker Green for 2 h after the RAW264.7 macrophage cells were incubated with 150 μg of DiDNPs or MM/DiDNPs for 4 h.

### Inflammatory cytokine assay in macrophages

The expression levels of typical inflammatory cytokines (tumor necrosis factor-α (TNF-α) and interleukin-6 (IL-6)) secreted by macrophages were determined. Specifically, RAW264.7 cells were seeded in 24-well plates at 1×10^5^ cells per well and cultured for 12 h. Then, the control group was treated with 100 ng/mL LPS. The other groups were first treated individually with free RAP, RAPNPs or MM/RAPNPs at various concentrations for 4 h and then stimulated with 100 ng/mL LPS for 24 h. Afterwards, the expression levels of TNF-α and IL-6 in the culture supernatant were determined by ELISA.

### Inhibition of proliferation of macrophages and SMCs *in vitro*

RAW264.7 cells and smooth muscle cells (SMCs) were seeded in a 96-well plate (10^4^ cells per well) and cultured in DMEM or 1640 medium containing 0.5% FBS for 12 h. Then, the cells were incubated with various doses of free RAP, RAP@PLGA, or RBC/RAP@PLGA for 24 h. Cell viability was quantified by MTS assay.

### Cell cytotoxicity evaluation

ECs, SMCs and RAW264.7 cells were seeded in 96-well plates at a density of 1.0×10^4^ cells per well in 100 μL of culture medium containing 10% (v/v) FBS, 100 U/mL penicillin, and 100 μg/mL streptomycin. Cells were incubated at 37 °C in a humidified atmosphere containing 5% CO_2_ for 12 h before the NPs were added. Then, the cells were treated with medium containing PLGA NPs or MM-coated PLGA NPs (MM/NPs) at various doses. After incubation for 24 h, cell viability was quantified by MTS assay.

### *In vitro* blood compatibility tests

The hemolysis of MM/RAPNPs was tested by a direct contact method *in vitro* as previously reported 50. Briefly, 1 mL of rabbit blood was diluted with 1.25 mL of 0.9% (w/v) sodium chloride solution. Then, 0.1 mL of the diluted whole blood sample was added to RAPNPs or MM/RAPNPs solution (5 mL, 1 mg/mL). Then, the specimens were continuously incubated at 37 °C for 1 h. Subsequently, the solutions were centrifuged for 5 min at 3000 rpm. The absorbance of the supernatant was measured at 540 nm using a microplate reader (μQuant, Bio-Tek Instruments Inc., Winooski, USA) to determine the released hemoglobin from lysed red blood cells. Untreated 0.9% (w/v) sodium chloride solution and double distilled water served as negative and positive controls.

The effect of MM/RAPNPs on platelet activation was also detected by measuring the concentration of platelet α granule membrane protein (GMP-140) in plasma after coincubation with nanoparticles. Briefly, anticoagulated whole rabbit blood was centrifuged at 1000 rpm for 10 min at 4 °C, and the supernatant plasma was collected. Then, 10 μL of 1.5 mg/mL RAPNP or MM/RAPNP solution was added to 300 μL of the prepared plasma, while saline was used as the control group. The samples were incubated at 37 °C for 30 min. After incubation, the concentration of GMP-140 in plasma was detected with an ELISA kit.

### Animals

Male C57BL/6 mice and male apolipoprotein E knockout (ApoE^-/-^) mice (eight weeks old) were obtained from the Third Military Medical University in Chongqing, China. Animals were housed in standard mouse cages with *ad libitum* access to water and food. Before experiments, all mice were acclimatized for at least 3 days. All animal-related procedures were in compliance with the China Council on Animal Care and Chongqing University protocol for animal use. All ethical guidelines for experimental animals were followed.

### *In vivo* long-term circulation test

The experiments were performed on adult male C57BL/6 mice weighing 25 ± 2 g. Briefly, DiDNPs and MM/DiDNPs were injected intravenously (200 μL, 2 mg/mL), and 30 μL of blood was rapidly collected from the tail after 1 min, 1 h, 6 h, 12 h, 24 h, 48 h. Blood samples were diluted with 30 μL of PBS containing EDTA-K2 in 96-well plates, and the fluorescence was measure with a microplate reader (TECAN M1000, USA) to determine fluorescence intensity.

### *In vivo* targeting to atherosclerotic plaques

ApoE**^-/-^** mice were fed a high-fat diet (HFD, consisting of a normal diet containing 0.5% cholesterol and 5% lard) for 2 months. DiDNPs and MM/DiDNPs were administered *via* the tail vein at a PLGA NP dosage of 2 mg/kg. After 24 h, the mice were euthanized and perfused with precooled PBS containing 4% paraformaldehyde to remove the blood and unbound nanoparticles. Each aorta from the root to the iliac bifurcation and the main organs were isolated for imaging and fluorescence quantification using an Xenogen IVIS 200 system. In addition, cross-sections of the aortic roots were observed by CLSM after staining with DAPI.

### Treatment of atherosclerosis in ApoE^-/-^ mice

ApoE**^-/-^** mice after 10 weeks of HFD feeding were randomized into 4 groups (5 mice per group) and dosed for 30 days by tail vein injection every three days. In the treatment groups, mice were administered free RAP, RAPNPs or MM/RAPNPs in 5% glucose at a dose of 0.7 mg/kg of RAP. Mice treated with only 5% glucose served as the model control group.

### Quantitative analysis of the atherosclerotic plaques

At the end stage of the treatment, the ApoE**^-/-^** mice were euthanized. Pathological evolution was evaluated by measuring the lesion area of atherosclerotic plaques in the aorta from the heart to the iliac bifurcation. Briefly, each aorta was fixed with paraformaldehyde (4% in PBS) for 1 h. After the periadventitial tissue was cleaned, the aorta was opened longitudinally, and then the entire aorta was stained with Oil red O (ORO) to quantify the plaque area. To determine the atherosclerotic extent at the aortic root, tissues embedded in the Tissue Tek® O.C.T. Compound (Sakura Finetek USA, Inc.) were cross-sectioned serially at 8 μm intervals and stained by ORO to quantify the area of the atherosclerotic plaques using Nis-Elements BR 3.2 software (Nikon, Japan).

### Histology and immunohistochemistry

The aortic sinus was fixed with paraformaldehyde (4% in PBS) for 1 h and then embedded in paraffin to cut into sections. After deparaffinizing and subsequently drying at 60 °C, sections were stained with toluidine blue to determine the necrotic core. For immunohistochemistry analysis, sections were immersed in 3% hydrogen peroxide and 100% methanol for 20 min to inhibit the activity of endogenous peroxidase and then blocked with 1% bovine serum albumin in PBS containing 0.3% Triton X-100 for 60 min. Antibodies for CD68, α-smooth muscle actin (α-SMA), and CD31 were coincubated for quantification of macrophages, SMCs, and ECs, respectively. The main organs, including the heart, liver, spleen, lung and kidney, were also harvested, fixed in paraformaldehyde (4% in PBS), and then sectioned for histology analysis by hematoxylin-eosin (H&E).

Tissue sample morphometry criteria for analyzing aortic cross-sections were based on previously described methods 51. For lipid deposition and necrotic core analysis, slides from 5 different mice per group were analyzed. For each ORO or toluidine blue stained slide, the vessel area, lipid deposition area and necrotic core areas were measured manually using ImageJ software. Additionally, the proportion of lipid area or necrotic core areas in each sample was calculated by dividing the vessel area by the lipid area or necrotic core areas. For the immunohistochemistry analyses of CD68 and α-SMA, slides from 5 different mice per group were analyzed. For each slide, the CD 68 or α-SMA positively stained cells within the atherosclerotic plaque areas were counted, and the plaque areas were measured using ImageJ software. The final cell count from each sample was divided by the plaque area to obtain a final cell density. All samples and groups were analyzed using the same parameters to maintain objectivity and eliminate bias. Lipid deposition and necrotic core percentage as well as the relative number of macrophages and SMCs were analyzed independently using 2-way repeated-measures ANOVA with a single pooled variance and Tukey's correction for pairwise comparisons within groups for each data set.

### Complete blood count and clinical chemistry

Blood was collected in EDTA spray-coated tubes and immediately analyzed for hematological parameters by an automated hematology analyzer (Sysmex KX-21, Sysmex Co., Japan), such as RBCs, platelets (PLTs), hemoglobin (HGB), white blood cells (WBCs), lymphocytes, monocytes and neutrophils. The plasma concentrations of alanine aminotransferase (ALT), aspartate aminotransferase (AST), alkaline phosphatase (ALP), creatinine (CREA), blood urea nitrogen (UREA), high-density lipoprotein (HDL), low-density lipoprotein (LDL), triglycerides (TGs) and total cholesterol (TC) were quantified by an automated analyzer platform (Roche Cobas C501, Roche Co., Switzerland).

### Statistical analysis

GraphPad Prism version 6.0 software (GraphPad, USA) was used for statistical analysis. Data analysis was performed using one-way analysis of variance (ANOVA). The minimum significance levels were set at *p < 0.05, **p < 0.01 and ***p < 0.001, with all data displayed as the mean ± SD.

## Results and Discussion

### Fabrication and characterization of MM/RAPNPs

**Figure 2:**
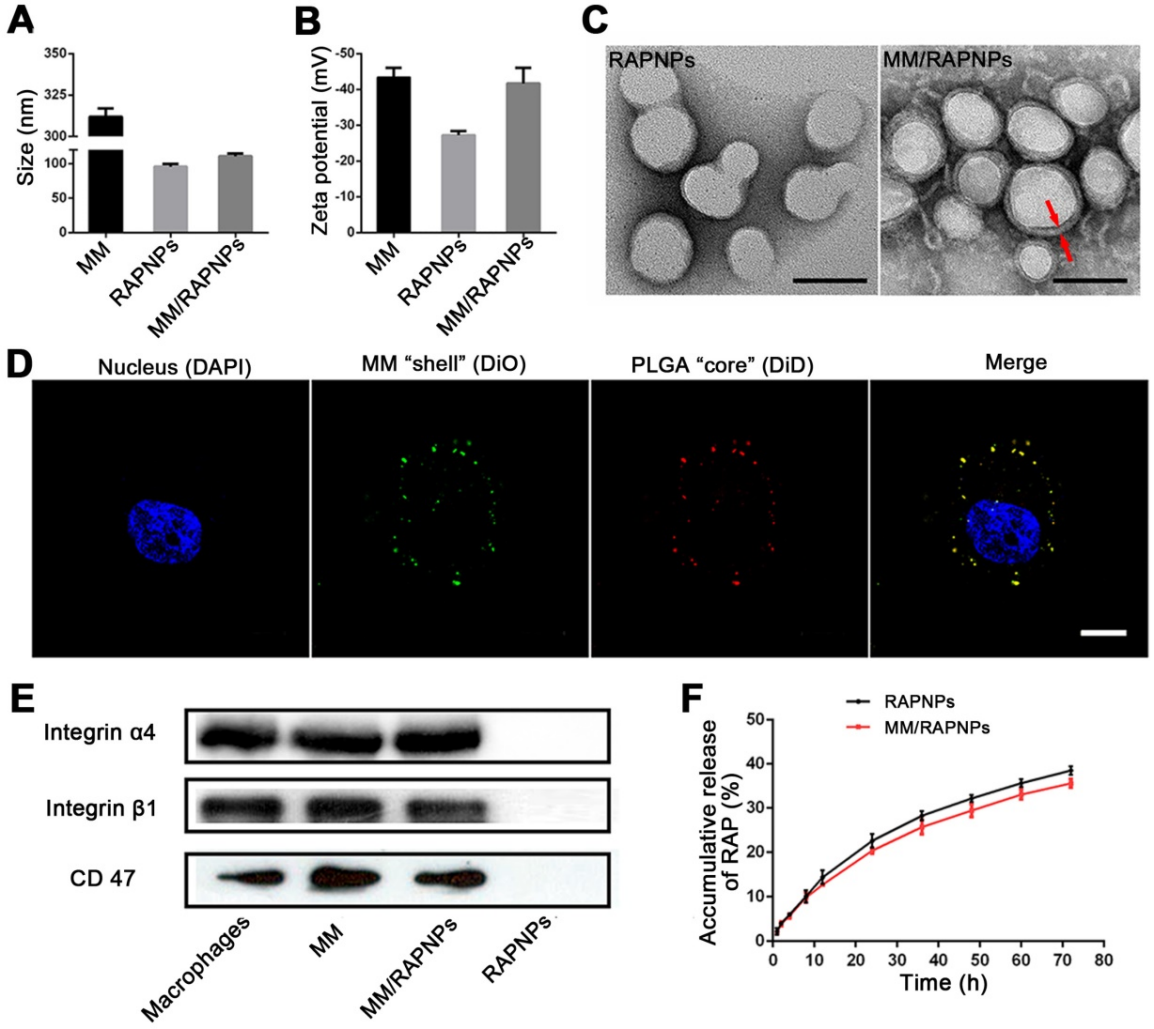
Characterization of the MM-coated biomimetic nanoparticles. (A) The sizes and (B) zeta potentials of MMs, RAPNPs and MM/RAPNPs (*n* = 3, mean ± SD). (C) TEM images of RAPNPs and MM/RAPNPs (scale bar = 100 nm). (D) CLSM images of the colocalization of the nucleus (blue), MM “shell” (green) and PLGA “core” (red) (scale bar = 5 µm). (E) Western blot results of integrin α4, integrin β1 and CD47 in macrophages, MMs, MM/RAPNPs and RAPNPs. (F) *In vitro* drug release profiles of RAPNPs and MM/RAPNPs (*n* = 3).

MM/RAPNPs were constructed using a three-step method: (i) preparation of the RAP-loaded PLGA nanoparticles (RAPNPs), (ii) isolation of the macrophage membrane (MM), and (iii) camouflage of the RAPNPs with the macrophage membrane. RAPNPs were first prepared by the nanoprecipitation method 47. Our results showed that the drug loading efficiency (LE) and encapsulation efficiency (EE) of RAPNPs were 6.87% and 76.3%, respectively (Table S1), indicating that hydrophobic RAP was efficiently encapsulated into the NPs. Dynamic light scattering (DLS) analysis indicated that the hydrodynamic diameter of RAPNPs was 95.69 nm with a favorable polydispersity index (PDI) of 0.110 (Figure 2A), and the zeta potential of RAPNPs was -26.4 mV (Figure 2B). Transmission electronic microscopy (TEM) measurements showed that the morphology of the RAPNPs particles was spherical with an average diameter of approximately 90 nm (Figure 2C). These results confirm that RAP-loaded PLGA nanoparticles are successfully prepared by the nanoprecipitation method.

To harvest MM/RAPNPs, RAPNPs were mixed with freshly prepared MM vesicles and subsequently extruded through a 200 nm porous polycarbonate membrane. Compared to uncoated RAPNPs, the hydrodynamic diameter of MM/RAPNPs increased from 95.69 to 110.8 nm, which was ascribed to the MM with a thickness of approximately 8 nm (Figure 2A). Additionally, the zeta potential of MM/RAPNPs (-41.7 mV) was comparable to that of the original MM (-43.4 mV) but much higher than that of the unmodified RAPNPs (-26.4 mV) (Figure 2B). The visual TEM image results confirmed that MM/RAPNPs showed a uniform “core-shell” structured morphology (Figure 2C). Furthermore, the single outer layer of the MM “shell” was approximately 8 nm thick, which agreed well with the previously reported thickness of the macrophage membrane 30,32. The MM coating on nanoparticles was also investigated by CLSM. Instead of RAP, the DiD fluorophore was loaded into the PLGA “core”, and the MM was labeled by DiO. As shown in Figure S1, the green membranes and red DiDNPs exhibited a high degree of colocalization, indicating the successful coating of MM on the DiDNPs. To further verify the stability of the “core-shell” structured nanoparticle, the fluorescently labeled NPs were incubated with HUVECs. The fluorescence images showed that the red fluorescence from DiD (representing the PLGA “core”) and the green fluorescence of DiO (representing the MM “shell”) colocalized well (Figure 2D), suggesting that MM/RAPNPs exhibited favorable stability even after cell internalization. After long-term storage at room temperature, MM/RAPNPs also showed a relatively constant size (Figure S2) in water and medium containing 10% FBS for 48 h, indicating satisfactory stability. In addition, the protein profiles in the MMs and MM/RAPNPs were determined by SDS-PAGE. The protein composition in the MM was mostly retained in the MM/RAPNPs, but no protein signal was detected from the RAPNPs (Figure S3), suggesting the successful translocation and retention of natural macrophage cell membranes onto the RAPNP surface. Moreover, due to the exclusive distribution of glycoproteins on the outside surface of the cell membranes, the orientation of the MM on the surface of the nanoparticles could be evaluated by quantification of the glycoproteins 49. As shown in Figure S4, the average glycoprotein content on MM/RAPNPs was approximately 92.95% of the amount in free MMs. This quantification suggests that the glycoproteins are strongly retained on the outside surface of the MM/RAPNPs, confirming their intrinsic “right-side-out” orientation when MMs are coated onto the nanoparticles. According to previous reports, the protein integrin α4β1 on the macrophage surface can specifically recognize and bind VCAM-1 30,40. Therefore, markers on macrophages, purified MMs, MM/RAPNPs and RAPNPs were detected by western blot measurements to determine the quality of the purified MMs and the effective decoration of the MMs on the MM/RAPNPs. The specific protein signals of integrin α4 and integrin β1 were observed in macrophages, MMs, and MM/RAPNPs, which validated the presence of these integrin markers (Figure 2E). In addition, the CD47 protein, which plays a key role in regulating macrophage phagocytosis by bonding with the SIRP-α receptor, was also detected 52. The results clearly showed that CD47 was retained on the MM and MM/RAPNPs (Figure 2E). Moreover, the protein signals of integrin α4, integrin β1 and CD47 were not detected in the RAPNPs. Collectively, this evidence suggests not only the successful decoration of RAPNPs with MMs to form MM/RAPNPs but also the retention of functional proteins to develop stealthy and targeted effects for potential advanced drug delivery.

The release kinetics of RAP from RAPNPs and MM/RAPNPs were investigated in buffer solutions that simulated the extracellular environment (PBS, pH 7.4). After 72 h of incubation, 38.51% and 35.62% of RAP was released from RAPNPs and MM/RAPNPs, respectively. Compared to RAPNPs, MM/RAPNPs showed a slightly slower RAP release profile (Figure 2F). In general, the steady and long-term RAP release behavior of MM/RAPNPs indicates their potential to be used for sustained drug release.

### Characterization of the immune-evasive functions and targeted delivery *in vitro*

**Figure 3:**
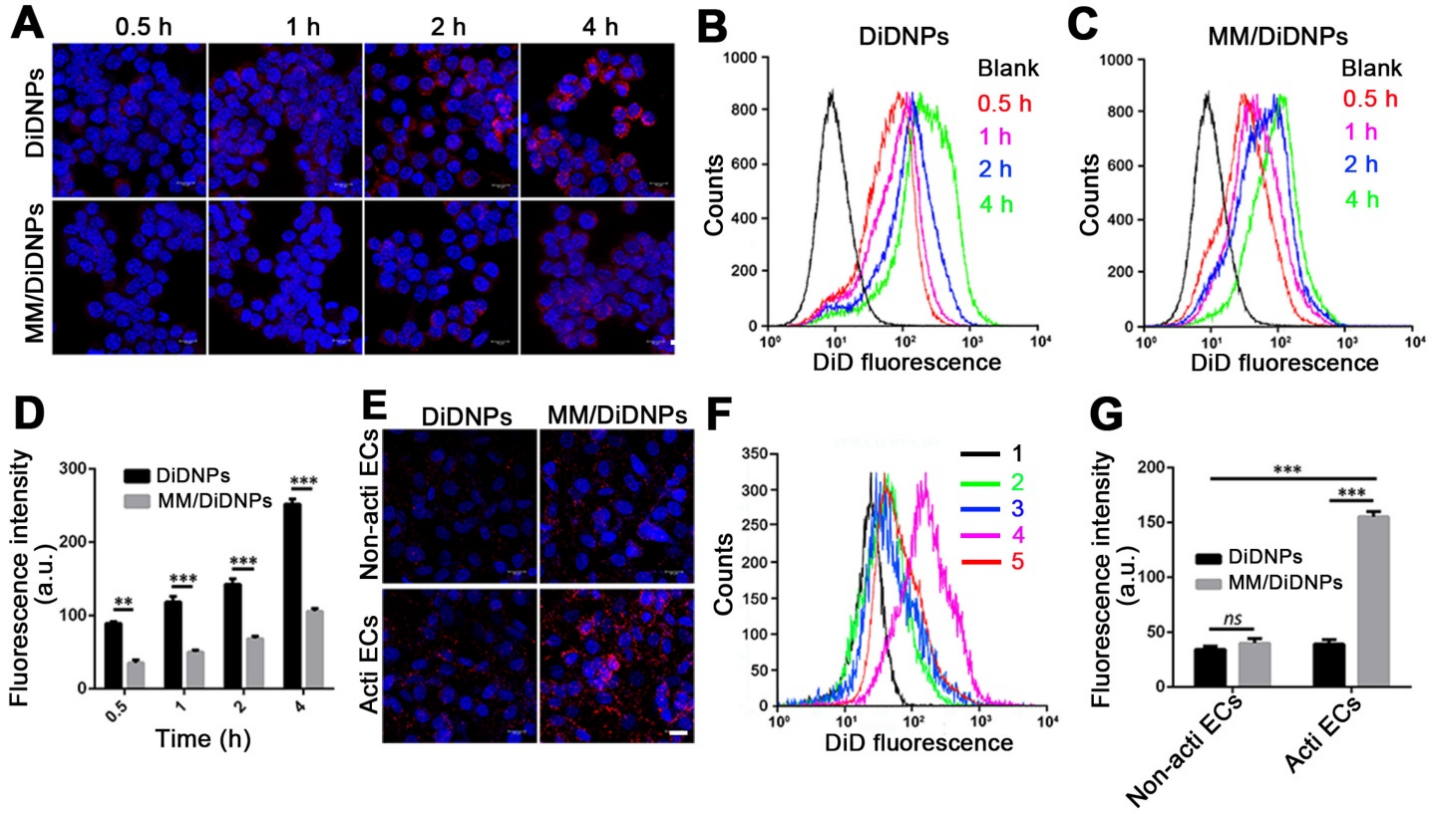
Cellular uptake by macrophages and HUVECs. (A) CLSM images of DiDNPs and MM/DiDNPs internalized by RAW264.7 cells (scale bar = 10 µm). FACS results of cellular uptake of (B) DiDNPs and (C) MM/DiDNPs in RAW264.7 cells. (D) Quantification of cellular uptake of DiDNPs and MM/DiDNPs in RAW264.7 cells (*n* = 3, mean ± SD). Cellular uptake of DiDNPs and MM/DiDNPs in HUVECs either in unactivated (Non-acti ECs) or activated (Acti-ECs) with tumor necrosis factor alpha (TNF-α) as demonstrated by (E) CLSM (scale bar = 20 µm), (F) FACS (1. blank; 2. Non-acti ECs + MM/DiDNPs; 3. Non-acti ECs + DiDNPs; 4. Acti ECs + MM/DiDNPs; 5. Acti ECs + DiDNPs ) and (G) fluorescent quantification of FACS (*n* = 3, mean ± SD). (**p < 0.01 and ***p < 0.001,* ns*, no significance.)

Accumulating evidence shows that MM-coated nanoparticles can inhibit phagocytosis by macrophage cells 30-32. The cellular phagocytosis of MM/DiDNPs was evaluated in RAW264.7 cells. The CLSM images showed that both DiDNPs and MM/DiDNPs were internalized by macrophages in a time-dependent manner. However, after internalization by macrophages, stronger red fluorescence from the DiDNPs was detected than the fluorescence from MM/DiDNPs at the same time (Figure 3A). This was further confirmed by FACS analysis using fluorescence quantification. After 0.5, 1, 2, and 4 h of incubation, the fluorescence-calculated internalization content of DiDNPs was approximately 2.5, 3.2, 2.0 and 2.4 times higher than that of the MM/DiDNPs, respectively (Figure 3B-D). In addition, staining of the lysosomes by LysoTracker (green fluorescence) revealed endolysosomal trafficking of most internalized DiDNPs and MM/DiDNPs in RAW264.7 cells because the red nanoparticles and green lysosomes exhibited a high degree of colocalization in both DiDNP- and MM/DiDNP-treated cells (Figure S5). The results demonstrate that the MM/DiDNPs can significantly inhibit internalization by macrophages, which is a great benefit to prolong their blood circulation time during bloodstream delivery by reducing undesirable clearance.

The decorated MMs influencing cellular uptake were evaluated using HUVECs. HUVECs activated with TNF-α could overexpress VCAM-1 26, ensuring the interaction specificity with integrinα4β1 on the macrophages. The cellular uptake of the DiDNPs and MM/DiDNPs was visually observed by CLSM and further quantified by FACS. The CLSM images showed that MM/DiDNPs displayed a higher internalization within activated endothelial cells compared with DiDNPs, showing stronger red fluorescence signals (Figure 3E). Moreover, FACS analysis showed that the cellular uptake of MM/DiDNPs in activated endothelial cells had a 3.0-fold higher signal than that of DiDNPs (Figure 3F, G). In addition, after using VCAM-1 antibodies to block VCAM-1 on activated HUVECs, the uptake of MM/DiDNPs by activated endothelial cells was obviously weakened. This indicates that VCAM-1 on HUVECs plays an important role in the interaction and cellular uptake of MM-coated NPs (Figure S6). In general, MM decoration on MM/DiDNPs enhanced the cellular uptake of MM/DiDNPs in activated endothelial cells, indicating a feasible strategy for targeted drug delivery in AS.

### *In vitro* cytotoxicity and blood compatibility

**Figure 4:**
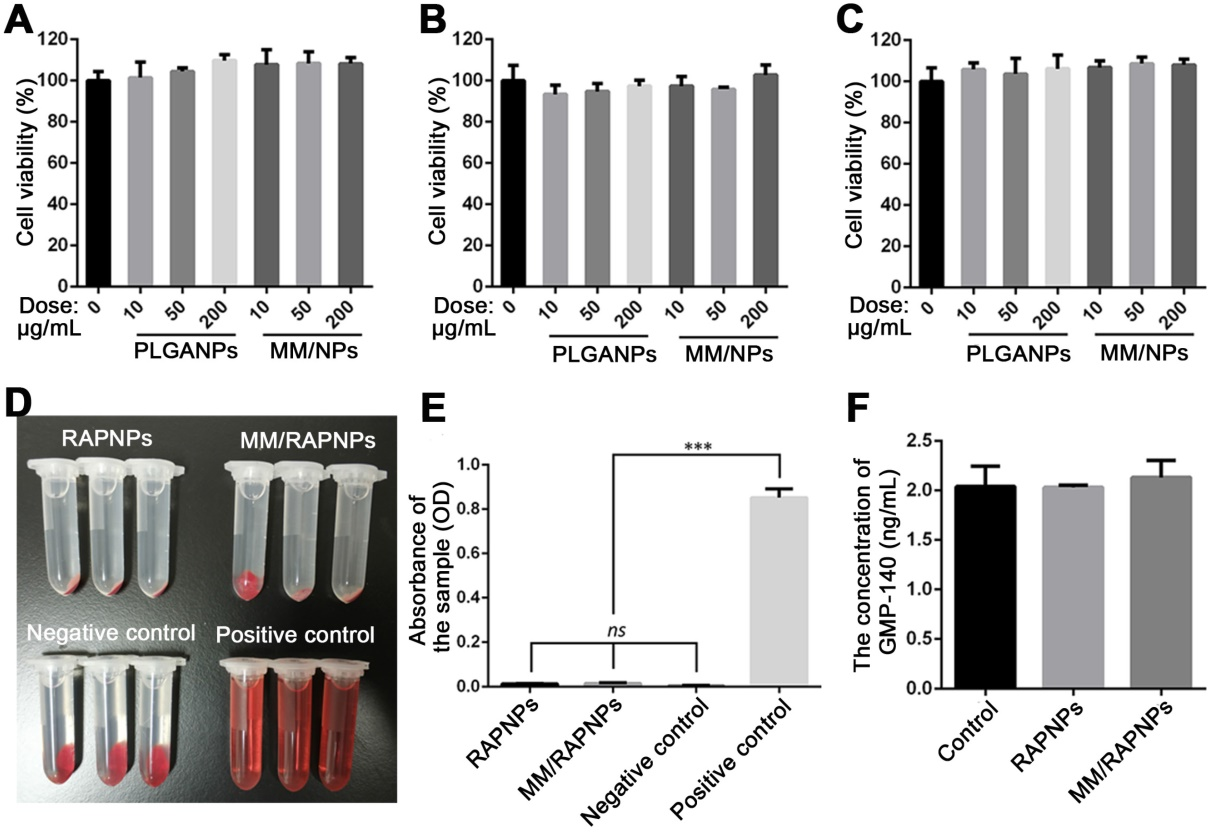
* In vitro* cytotoxicity and blood compatibility studies. Cell viability of (A) ECs, (B) SMCs and (C) RAW264.7 cells after incubation with various doses of NPs for 24 h. (D) Images of the hemolysis test with RAPNPs and MM/RAPNPs. (E) The absorbance of RAPNPs and MM/RAPNPs measured at 540 nm (*n* = 3, mean ± SD). (***p < 0.001; *ns*, no significance). (B) The concentration of platelet α-granule membrane protein (GMP-140) in plasma after incubation with different samples (*n* = 3, mean ± SD.)

Subsequently, we evaluated the *in vitro* biological effects of MM-coated nanoparticles. The cytotoxicity of PLGA NPs and MM-coated NPs in ECs, SMCs and RAW264.7 cells was investigated. As shown in Figure 4A-C, after 24 h of incubation with PLGA NPs and MM/NPs at the doses of 10, 50, or 200 μg/mL, no significant changes in cell viability were observed compared to the control. These results suggested that both PLGA NPs and MM/NPs exhibited good cytocompatibility.

Blood compatibility is an important safety index of biomaterials, especially those that are in direct contact with blood 53. Therefore, we tested the blood compatibility of MM/RAPNPs *in vitro*. First, the hemolysis of MM/RAPNPs was detected by the direct contact method. The visual hemolytic images showed no significant hemolysis of either RAPNPs or MM/RAPNPs at a concentration of 1 mg/mL (Figure 4D). The OD values of RAPNPs and MM/RAPNPs were not significantly different from those of the negative control group (Figure 4E). The results showed that RAPNPs and MM/RAPNPs are nonhemolytic.

Furthermore, the effects of MM/RAPNPs on platelet activation were also detected by measuring the concentration of GMP-140 in plasma after coincubation with NPs. The concentration of GMP-140 in the RAPNP and MM/RAPNP groups showed little difference compared with the negative control group (Figure 4F), indicating that RAPNPs and MM/RAPNPs were safe enough to avoid undesirable platelet activation. Therefore, the results confirmed that both RAPNPs and MM/RAPNPs have good blood compatibility.

### *In vitro* antiatherosclerotic effects

The proinflammatory cytokines secreted by macrophages are the primary factor involved in the pathogenesis of atherosclerosis 35. Accordingly, the capability of NPs to alleviate the expression of the inflammatory cytokines in macrophages was determined *in vitro*. As shown in Figure S7, the levels of typical proinflammatory cytokines, including TNF-α and IL-6, were remarkably downregulated in the cells treated with RAPNPs or MM/RAPNPs, which was attributed to the satisfactory anti-inflammatory effects of RAP. Moreover, since the proliferation of macrophages and SMCs plays an important role in atherosclerosis progression 37,54, we examined whether RAP-loaded nanoparticles inhibit the proliferation of RAW264.7 cells and SMCs *in vitro*. As shown in Figure S8, RAP inhibited the viability of macrophages and SMCs in a dose-dependent manner. Moreover, at the same dose, RAPNPs and MM/RAPNPs showed comparable inhibition of cell proliferation. The slightly more potent antiproliferative activity of free RAP might be ascribed to the slower RAP release from RAPNPs and MM/RAPNPs. Collectively, these data demonstrated that MM/RAPNPs can attenuate LPS-induced inflammation and inhibit the proliferation of macrophages and SMCs, suggesting the significant potential of these biomaterials for atherosclerosis therapy.

### *In vivo* targeting of atherosclerotic plaques

**Figure 5:**
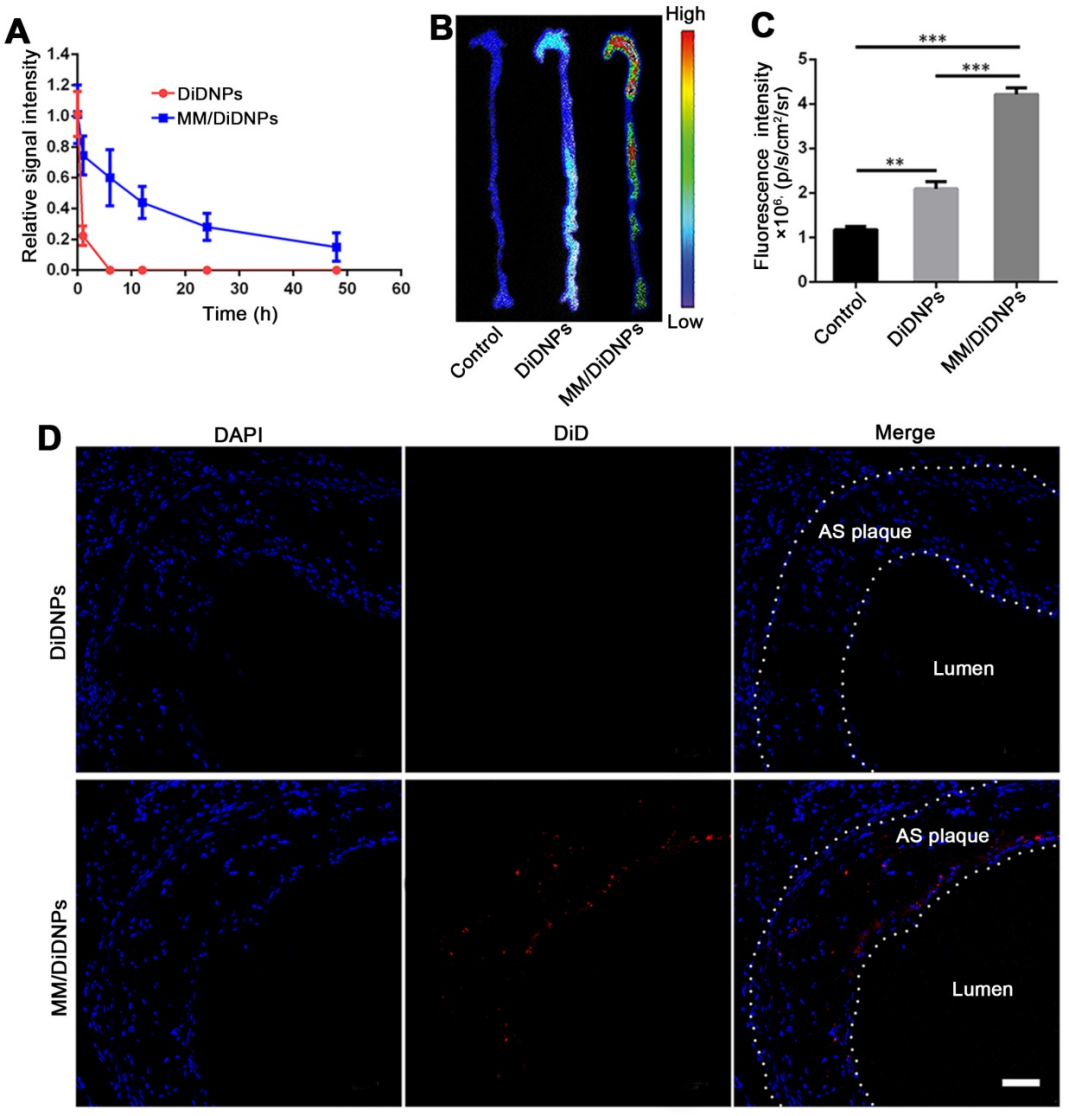
Targeting atherosclerotic plaques in ApoE^-/-^ mice. (A) Relative fluorescence intensity of DiDNPs and MM/DiDNPs in blood. (B) Representative *ex vivo* fluorescence images and (C) quantitative data of DiD fluorescent signals accumulated in the aorta 24 h postinjection (*n* =3, mean ± SD, **p < 0.01 and ***p < 0.001, *ns*, no significance). (D) CLSM images of accumulated MM/DiDNPs in atherosclerotic plaques of an aortic root section in ApoE^-/-^ mice (AS plaque outlined by white dashed line, scale bar = 60 µm.)

To assess whether MM/DiDNPs inherited a long circulation lifetime from the natural MMs, we studied the pharmacokinetics *in vivo* in a C57BL/6 mouse model. After intravenous injection *via* the tail vein, the residual content of the nanomedicine was evaluated by measuring the relative signal intensity of the collected blood at certain time interval using fluorescence spectroscopy. Compared with the bare DiDNPs, the MM/DiDNPs could significantly enhance the blood retention time over a span of 48 h. Interestingly, more than 15% of MM/DiDNPs were retained in blood vessels even after 48 h of blood circulation, whereas the bare DiDNPs were almost eliminated from the blood at 8 h postinjection (Figure 5A). Therefore, the MM/DiDNPs exhibited superior blood retention, suggesting that the immunosuppressive surface makeup of the MM is able to efficiently prolong the blood circulation time to potentially enhance targeted drug delivery for AS.

The targeting ability of the MM/DiDNPs to the atherosclerotic regions was assessed in the ApoE^-/-^ mouse model. After 24 h of intravenous administration of the DiD-loaded nanoparticles, the mice were sacrificed, and their main organs and aortas were harvested and processed *ex vivo*. The MM/DiDNPs accumulated in the atherosclerotic plaques could be clearly observed by *ex vivo* imaging. Strong fluorescence was found in the regions of the aortic arc, a region prone to developing atherosclerosis (Figure 5B). By contrast, the DiDNP group showed relatively weak fluorescence in the atherosclerotic plaque regions, which was much lower than that of the MM/DiDNP group (Figure 5C). In addition, the fluorescence images of the cross-sections from the plaque regions showed that accumulated MM/DiDNPs were largely localized within plaque regions (Figure 5D). This result demonstrated that MM functionalization could enhance MM/DiDNP accumulation within plaque regions *in vivo*. In addition, at 24 h postinjection, the fluorescence signals were mainly distributed in the liver, kidney and lung (Figure S9A). The fluorescence signals in the liver and kidney of the MM/DiDNPs group were significantly lower than those in the DiDNPs group (Figure S9B). This result confirms that NPs coated with MMs can reduce the accumulation of NPs in the main organs *in vivo*, which could reduce the nonspecific toxicity and side effects of MM-coated NPs.

It is well documented that dysfunctional endothelium is the main pathological feature in atherosclerotic areas. Specifically, dysfunctional endothelial cells express adhesion molecules (e.g., VCAM-1) and secrete chemokines to recruit circulating monocyte macrophages and promote them to traverse the subendothelial space and migrate into the intimal layer 55,56. In addition, macrophages are large, highly versatile white blood cells that intrinsically work as major cellular effectors in inflammation and tissue repair processes 28,57. There are proteins (e.g., integrin α4β1) on the surface of macrophage membranes that can interact with dysfunctional endothelial cells through integrin-mediated adhesive interactions, such as the integrin α4β1/VCAM-1 interaction 30,40. Therefore, MM-coated NPs that inherit functional proteins of macrophage membranes can actively target dysfunctional endothelium. Moreover, the EPR effect also exists in atherosclerotic lesions based on the leaky endothelium from inflammation and the leaky microvessels in atherosclerotic plaques, which allows nanoparticles to permeate the vascular wall and accumulate within the pathological lesion 58,59. Overall, MM-coated NPs with long circulation and specific interactions with dysfunctional endothelium have the ability to efficiently target and accumulate in atherosclerotic lesions.

### *In vivo* therapeutic efficacy

**Figure 6:**
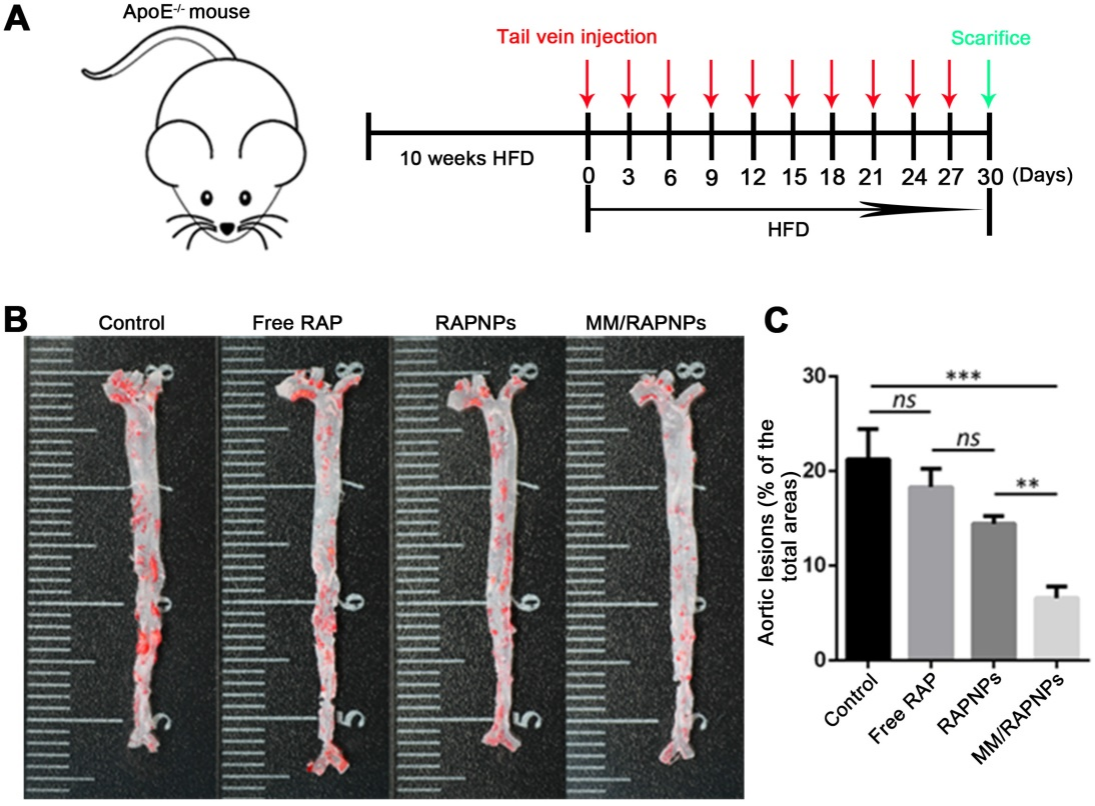
Therapeutic efficacy of atherosclerosis in ApoE^-/-^ mice. (A) Schematic of the experimental design in this study. (B) Representative photographs of *en face* ORO-stained aortas. (C) Quantitative analysis of the lesion area (*n* = 5*,* mean ± SD). (**p < 0.01, ***p < 0.001 and *ns*, no significance.)

The influence of MM/RAPNPs on AS development was analyzed in an AS pathological model in ApoE^-/-^ mice. After 30 days of treatment, the aortas were isolated and stained with ORO (Figure 6A). The *en face* micrographs of ORO-stained aortas showed that MM/RAPNP treatment potently inhibited the progression of atherosclerotic lesions in ApoE^-/-^ mice (Figure 6B and Figure S10). To quantitatively evaluate the atherosclerotic lesion, the lesion-to-aorta area ratio was calculated. As shown in Figure 5C, there were no significant therapeutic effects from free RAP treatment compared with the control group. It is well known that RAP, a biopharmaceutical classification system (BCS) class II drug, is practically insoluble in water 44. Its poor solubility resulted in the low bioavailability when administered by tail vein injection. Compared with free RAP and RAPNPs, which resulted in 18.3% and 14.43% atherosclerotic lesions, respectively, MM/RAPNPs yielded significantly lower atherosclerotic lesions (6.59%) (Figure 6C).

**Figure 7:**
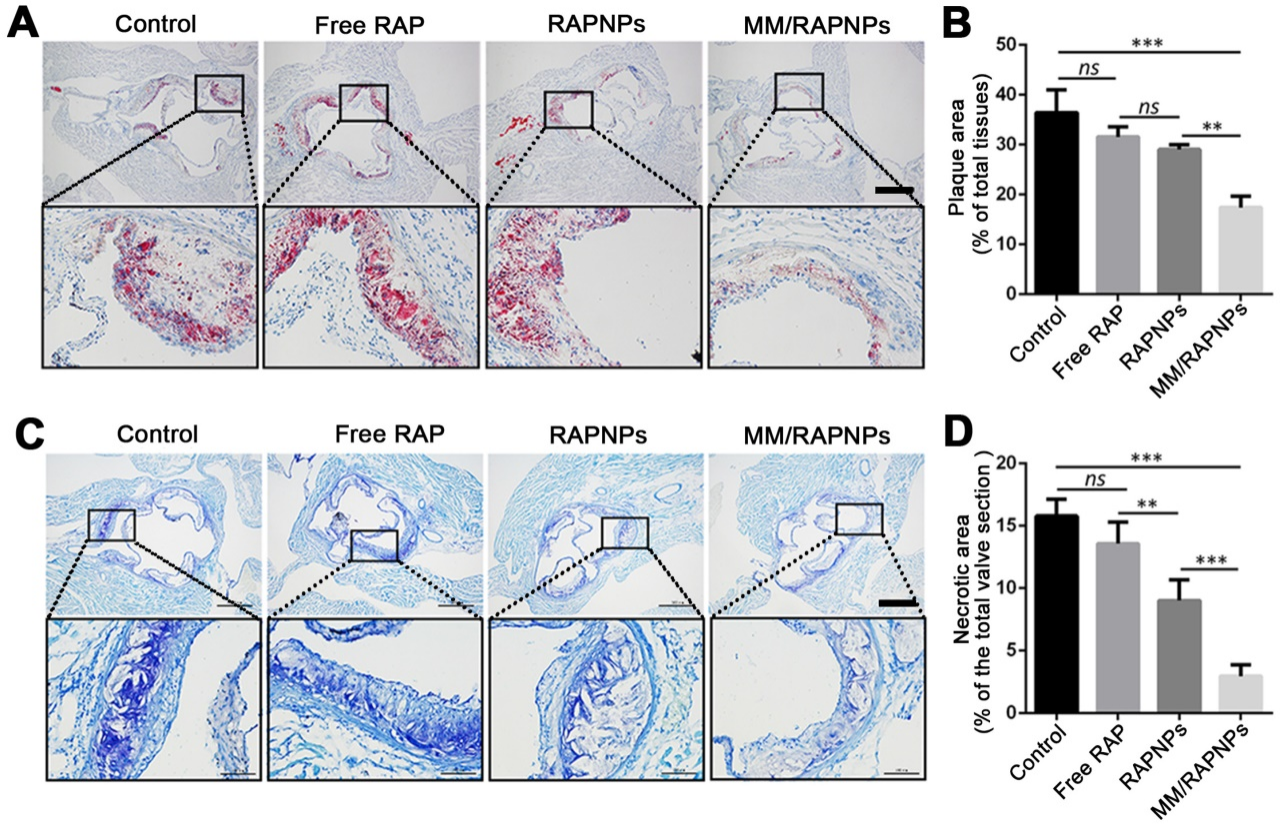
(A) ORO-stained cross-sections of aortic roots (scale bar = 500 µm). (B) Quantitative analysis of the lipid deposition area in the cross-sections of the aortic root (*n* = 5*,* mean ± SD). (C) The necrotic core areas stained by toluidine blue (scale bar = 500 µm). (D) Quantitative analysis of the necrotic cores of plaque lesions in cross-sections of the aortic root (*n* = 5*,* mean ± SD). (**p < 0.01, ***p < 0.001 and* ns*, no significance.)

To further investigate lipid deposition and the formation of necrotic areas in atherosclerotic plaques, cross-sections of the aortic root were stained with ORO and toluidine blue, respectively. According to the ORO-stained cross-sections of the aortic root, a large amount of lipids (up to 36.45%) were deposited in the plaques of the control group (Figure 7A, B). Compared with the control group (5% glucose), the extent of lipid deposition was reduced in the free RAP and RAPNP groups (31.54% and 29.05%, respectively). Most notably, after treatment with MM/RAPNPs, a reduced amount of lipids (17.41%) were found in the plaques (Figure 7A, B). Furthermore, toluidine blue staining showed that large acellular cores and massive cholesterol crystals were found in the plaques of the control group, with an average area of necrotic cores of 15.79% (Figure 7C, D). The average area of necrotic cores decreased to 13.57%, 9.00% and 2.95% in the free RAP, RAPNP and MM/RAPNP treatment groups, respectively (Figure 7C, D). These results reveal that MM/RAPNPs can effectively attenuate the progression of atherosclerosis.

**Figure 8:**
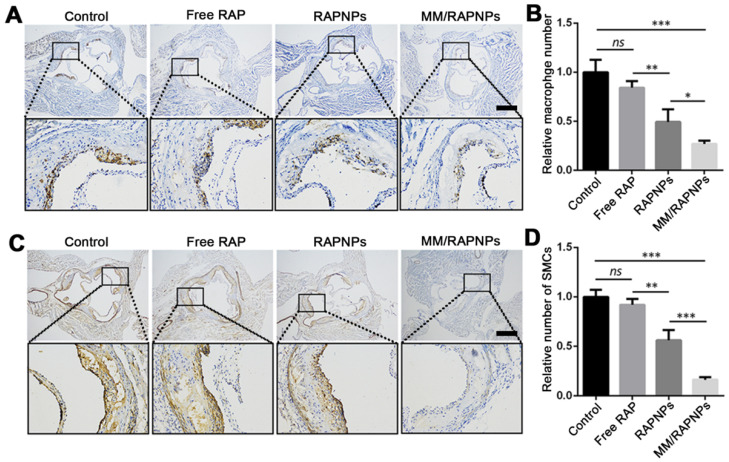
Representative immunohistochemistry staining photographs with antibodies against (A) CD68 and (C) α-SMA (scale bar = 500 µm). Quantitative analysis the relative number of (B) macrophages and (D) SMCs in plaque lesions of cross-sections of the aortic root (*n* = 5*,* mean ± SD). (*p < 0.05, **p < 0.01, ***p < 0.001 and* ns*, no significance.)

To further study the therapeutic mechanism of MM/RAPNPs, macrophages and SMCs in atherosclerotic lesion areas were investigated by immunohistochemistry. Previous studies have reported that the abnormal proliferation of macrophages and SMCs promotes the progression of atherosclerosis 37,54. Immunohistochemistry analyses for CD68 (macrophage marker) (Figure 8A, B) and α-SMA (SMC marker) (Figure 8C, D) showed that the number of macrophages and SMCs dramatically decreased in aortic root sections, particularly in the MM/RAPNP-treated group. Immunohistochemistry analyses for CD31 (a marker for ECs) showed notable expression of CD31 in the vascular endothelium of the aortas from mice treated with MM/RAPNPs (Figure S11), which indicated that MM/RAPNP treatment might maintain the integrity of the vascular endothelium. These results substantiated that MM/RAPNPs could significantly inhibit the growth of macrophages and SMCs in atherosclerotic areas to delay the progression of AS. The above results demonstrate the remarkable efficacy of MM/RAPNPs in targeting AS treatment. In general, the macrophage membrane coating strategy endows MM/RAPNPs with the functions of long-term circulation and active targeting to the dysfunctional endothelium, allowing MM/RAPNPs to efficiently accumulate at atherosclerotic lesions. Then, the loaded RAP is released from the MM/RAPNPs, thereby increasing the local drug concentration to inhibit the proliferation of macrophages and SMCs and the inflammatory responses in the lesion, finally significantly attenuating the progression of atherosclerosis.

### Biosafety assessment

**Figure 9:**
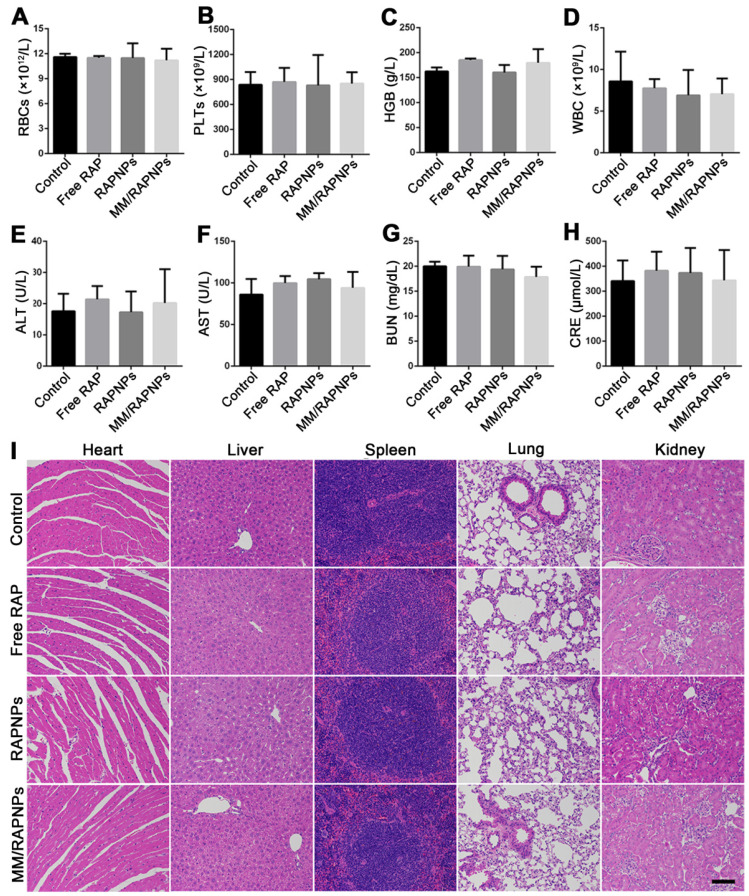
Preliminary safety evaluation. (A-D) Typical hematological parameters (*n* = 5*,* mean ± SD). (E-H) Biochemical markers relevant to hepatic and kidney function (*n* = 5*,* mean ± SD). (I) H&E stained sections of major organs resected from mice subjected to treatment with various formulations for one month (scale bar = 100 µm).

During drug treatment, toxic side effects to normal organs and the whole system of nanoparticles have been major problems 60. To assess biosafety, the potential side effects were investigated after one month of treatment. Complete blood count implied that red blood cells (RBCs), platelets (PLTs), and hemoglobin (HGB) displayed no significant variations (Figure 9A-C). Specifically, the counts of immune-associated cells, including white blood cells (WBCs), monocytes, lymphocytes and neutrophils, in the blood of the treated mice were similar to those of the mice in the control group (Figure 9D and Figure S12). Clinical biochemistry analysis showed normal levels of alanine aminotransferase (ALT), aspartate aminotransferase (AST), blood urea nitrogen (BUN) and creatinine (CRE), indicating that the biological functions of the liver and kidney were not affected by treatment (Figure 9E-H). Additionally, the levels of high-density lipoprotein cholesterol (HDL), low-density lipoprotein cholesterol (LDL), total cholesterol (TC) and triglycerides (TGs) were not significantly altered during the treatment (Figure S13). The results of H&E staining also indicated that no significant changes or injuries could be found in the main organs, further confirming their biocompatibility (Figure 9I). Accordingly, MM/RAPNPs had no obvious immunotoxicity or side effects after long-term treatment, making MM/RAPNPs a safe potential candidate for chronic vascular disease therapy. Moreover, in our study, RAW264.7 cells were used as a model macrophage cell line to extract membranes, and the* in vitro* and *in vivo* experimental results supported the validity of this model. However, primary macrophages such as bone marrow-derived macrophages might act as a better macrophage model and membrane source to fabricate macrophage-based biomimetic drug delivery systems.

## Conclusions

In this study, we developed a biomimetic targeted nanoparticle to efficiently and safely inhibit the progression of atherosclerosis. In our nanoformulation, MMs were coated onto RAPNPs, which showed favorable sustained drug release kinetics, effectively inhibited macrophage phagocytosis and targeted activated endothelial cells *in vitro*. In the AS mouse model, MM-camouflaged NPs can efficiently accumulate in atherosclerotic plaques. Additionally, *in vivo* therapy results illustrate that MM/RAPNPs significantly delayed the progression of atherosclerosis after treatment for one month. Finally, the biomimetic nanoparticles displayed a good safety profile without any significant side effects after long-term administration in mice. Therefore, these MM-functionalized biocompatible NPs represent new potential nanocarriers that hold considerable promise as an effective targeted drug delivery system to treat atherosclerosis.

## Supplementary Material

Supplementary figures and tables.

## Abbreviations

AS: atherosclerosis
PLGA: poly (lactic-co-glycolic acid) copolymer
CVDs: cardiovascular diseases
NPs: nanoparticles
RBC: red blood cell
VCAM-1: vascular cell adhesion molecule-1
RAP: rapamycin
mTOR: the mammalian target of RAP
BCA: bicinchoninic acid
MWCO: molecular weight cut-off
TEM: transmission electron microscopy
LE: loading efficiency
EE: encapsulation efficiency
PBS: phosphate-buffered saline
HUVECs: human umbilical vein endothelial cells
FBS: fetal bovine serum
DAPI: 4′,6-diamidino-2-phenylindole
FACS: fluorescence-activated cell sorting
CLSM: confocal laser scanning microscopy
TNF-α: tumor necrosis factor-α
IL-6: interleukin-6
GMP-140: platelet α granule membrane protein
ApoE^-/-^: apolipoprotein E knockout
HFD: high-fat diet
ORO: Oil red O
α-SMA: α-smooth muscle actin
H&E: hematoxylin-eosin
PLTs: platelets
HGB: hemoglobin
WBCs: white blood cells
ALT: alanine aminotransferase
AST: aspartate aminotransferase
ALP: alkaline phosphatase
CREA: creatinine
UREA: blood urea nitrogen
HDL: high-density lipoprotein
LDL: low-density lipoprotein
TGs: triglycerides
TC: total cholesterol
PDI: polydispersity index

## References

[1] Wang T, Butany J (2017). Pathogenesis of atherosclerosis. Diagn Histopathol.

[2] Libby P (2002). Inflammation in atherosclerosis. Nature.

[3] Raggi P, Genest J, Giles JT, Rayner KJ, Dwivedi G, Beanlands RS (2018). Role of inflammation in the pathogenesis of atherosclerosis and therapeutic interventions. Atherosclerosis.

[4] Herrington W, Lacey B, Sherliker P, Armitage J, Lewington S (2016). Epidemiology of atherosclerosis and the potential to reduce the global burden of atherothrombotic disease. Circ Res.

[5] Adhyaru BB, Jacobson TA (2018). Safety and efficacy of statin therapy. Nat Rev Cardiol.

[6] Kim D, Shin K, Kwon SG, Hyeon T (2018). Synthesis and biomedical applications of multifunctional nanoparticles. Adv Mater.

[7] Zhou L, Wang H, Li Y (2018). Stimuli-responsive nanomedicines for overcoming cancer multidrug resistance. Theranostics.

[8] Yohan D, Chithrani BD (2014). Applications of nanoparticles in nanomedicine. J Biomed Nanotechnol.

[9] Chan C, Zhang L, Cheng CK, Yang H, Huang Y, Tian XY (2018). Recent advances in managing atherosclerosis via nanomedicine. Small.

[10] Wang Y, Li L, Zhao W, Dou Y, An H, Tao H (2018). Targeted therapy of atherosclerosis by a broad-spectrum reactive oxygen species scavenging nanoparticle with intrinsic anti-inflammatory activity. ACS Nano.

[11] Li C, Dou Y, Chen Y, Qi Y, Li L, Han S Site-specific microRNA-33 antagonism by pH-responsive nanotherapies for treatment of atherosclerosis via regulating cholesterol efflux and adaptive immunity. Adv Funct Mater. 2020: 2002131.

[12] Cheng J, Zhang R, Li C, Tao H, Dou Y, Wang Y (2018). A Targeting nanotherapy for abdominal aortic aneurysms. J Am Coll Cardiol.

[13] Liu J, Li M, Luo Z, Dai L, Guo X, Cai K (2017). Design of nanocarriers based on complex biological barriers *in vivo* for tumor therapy. Nano Today.

[14] Blanco E, Shen H, Ferrari M (2015). Principles of nanoparticle design for overcoming biological barriers to drug delivery. Nat Biotechnol.

[15] Nie S (2010). Understanding and overcoming major barriers in cancer nanomedicine. Nanomedicine (Lond).

[16] Ai X, Hu M, Wang Z, Zhang W, Li J, Yang H (2018). Recent advances of membrane-cloaked nanoplatforms for biomedical applications. Bioconjug Chem.

[17] Fang RH, Kroll AV, Gao W, Zhang L (2018). Cell membrane coating nanotechnology. Adv Mater.

[18] Zhang P, Liu G, Chen X (2017). Nanobiotechnology: Cell membrane-based delivery systems. Nano Today.

[19] Yan H, Shao D, Lao YH, Li M, Hu H, Leong KW (2019). Engineering cell membrane-based nanotherapeutics to target inflammation. Adv Sci.

[20] Tan S, Wu T, Zhang D, Zhang Z (2015). Cell or cell membrane-based drug delivery systems. Theranostics.

[21] Parodi A, Quattrocchi N, van de Ven AL, Chiappini C, Evangelopoulos M, Martinez JO (2013). Synthetic nanoparticles functionalized with biomimetic leukocyte membranes possess cell-like functions. Nat Nanotechnol.

[22] Zhang Q, Dehaini D, Zhang Y, Zhou J, Chen X, Zhang L (2018). Neutrophil membrane-coated nanoparticles inhibit synovial inflammation and alleviate joint damage in inflammatory arthritis. Nat Nanotechnol.

[23] Zhuang J, Gong H, Zhou J, Zhang Q, Gao W, Fang RH (2020). Targeted gene silencing *in vivo* by platelet membrane-coated metal-organic framework nanoparticles. Sci Adv.

[24] Park JH, Dehaini D, Zhou J, Holay M, Fang RH, Zhang L (2020). Biomimetic nanoparticle technology for cardiovascular disease detection and treatment. Nanoscale Horiz.

[25] Song Y, Huang Z, Liu X, Pang Z, Chen J, Yang H (2019). Platelet membrane-coated nanoparticle-mediated targeting delivery of rapamycin blocks atherosclerotic plaque development and stabilizes plaque in apolipoprotein E-deficient (ApoE(-/-)) mice. Nanomedicine-UK.

[26] Wei X, Ying M, Dehaini D, Su Y, Kroll AV, Zhou J (2018). Nanoparticle functionalization with platelet membrane enables multifactored biological targeting and detection of atherosclerosis. ACS Nano.

[27] Wang Y, Zhang K, Qin X, Li T, Qiu J, Yin T (2019). Biomimetic nanotherapies: red blood cell based core-shell structured nanocomplexes for atherosclerosis management. Adv Sci.

[28] Watanabe S, Alexander M, Misharin AV, Budinger G (2019). The role of macrophages in the resolution of inflammation. J Clin Invest.

[29] Hamidzadeh K, Christensen SM, Dalby E, Chandrasekaran P, Mosser DM (2017). Macrophages and the recovery from acute and chronic inflammation. Annu Rev Physiol.

[30] Cao H, Dan Z, He X, Zhang Z, Yu H, Yin Q (2016). Liposomes coated with isolated macrophage membrane can target lung metastasis of breast cancer. ACS Nano.

[31] Xuan M, Shao J, Dai L, Li J, He Q (2016). Macrophage cell membrane camouflaged Au nanoshells for *in vivo* prolonged circulation life and enhanced cancer photothermal therapy. ACS Appl Mater Interfaces.

[32] Xuan M, Shao J, Dai L, He Q, Li J (2015). Macrophage cell membrane camouflaged mesoporous silica nanocapsules for *in vivo* cancer therapy. Adv Healthc Mater.

[33] Thamphiwatana S, Angsantikul P, Escajadillo T, Zhang Q, Olson J, Luk BT (2017). Macrophage-like nanoparticles concurrently absorbing endotoxins and proinflammatory cytokines for sepsis management. Proc Natl Acad Sci U S A.

[34] Li R, He Y, Zhu Y, Jiang L, Zhang S, Qin J (2019). Route to rheumatoid arthritis by macrophage-derived microvesicle-coated nanoparticles. Nano Lett.

[35] Bobryshev YV, Nikiforov NG, Elizova NV, Orekhov AN (2017). Macrophages and their contribution to the development of atherosclerosis. Results Probl Cell Differ.

[36] Lu X (2016). Impact of macrophages in atherosclerosis. Curr Med Chem.

[37] Moore KJ, Sheedy FJ, Fisher EA (2013). Macrophages in atherosclerosis: a dynamic balance. Nat Rev Immunol.

[38] Woollard KJ, Geissmann F (2010). Monocytes in atherosclerosis: subsets and functions. Nat Rev Cardiol.

[39] Wu CH, Daugherty A, Lu H (2018). Multifaceted functions of macrophages in atherosclerosis. Curr Opin Lipidol.

[40] Tang TT, Lv LL, Wang B, Cao JY, Feng Y, Li ZL (2019). Employing macrophage-derived microvesicle for kidney-targeted delivery of dexamethasone: an efficient therapeutic strategy against renal inflammation and fibrosis. Theranostics.

[41] Gao C, Huang Q, Liu C, Kwong C, Yue L, Wan JB (2020). Treatment of atherosclerosis by macrophage-biomimetic nanoparticles via targeted pharmacotherapy and sequestration of proinflammatory cytokines. Nat Commun.

[42] Kennedy BK, Lamming DW (2016). The mechanistic target of rapamycin: the grand conducTOR of metabolism and aging. Cell Metab.

[43] Dou Y, Chen Y, Zhang X, Xu X, Chen Y, Guo J (2017). Non-proinflammatory and responsive nanoplatforms for targeted treatment of atherosclerosis. Biomaterials.

[44] Dou Y, Zhang X, Xu X, Zhou X, Han S, Wang R (2015). Multiple noncovalent interactions mediated one-pot therapeutic assemblies for the effective treatment of atherosclerosis. J Mater Chem B.

[45] Chen WQ, Zhong L, Zhang L, Ji XP, Zhang M, Zhao YX (2009). Oral rapamycin attenuates inflammation and enhances stability of atherosclerotic plaques in rabbits independent of serum lipid levels. Br J Pharmacol.

[46] Li SY, Cheng H, Xie BR, Qiu WX, Zeng JY, Li CX (2017). Cancer cell membrane camouflaged cascade bioreactor for cancer targeted starvation and photodynamic therapy. ACS Nano.

[47] Zhuang J, Fang RH, Zhang L (2017). Preparation of particulate polymeric therapeutics for medical applications. Small Methods.

[48] Hu CM, Fang RH, Wang KC, Luk BT, Thamphiwatana S, Dehaini D (2015). Nanoparticle biointerfacing by platelet membrane cloaking. Nature.

[49] Luk BT, Hu CM, Fang RH, Dehaini D, Carpenter C, Gao W (2014). Interfacial interactions between natural RBC membranes and synthetic polymeric nanoparticles. Nanoscale.

[50] Luo L, Wu W, Sun D, Dai HB, Wang Y, Zhong Y (2018). Acid-activated melittin for targeted and safe antitumor therapy. Bioconjug Chem.

[51] Boada C, Zinger A, Tsao C, Zhao P, Martinez JO, Hartman K (2020). Rapamycin-loaded biomimetic nanoparticles reverse vascular inflammation. Circ Res.

[52] Rodriguez PL, Harada T, Christian DA, Pantano DA, Tsai RK, Discher DE (2013). Minimal "Self" peptides that inhibit phagocytic clearance and enhance delivery of nanoparticles. Science.

[53] Bender EA, Adorne MD, Colomé LM, Abdalla DSP, Guterres SS, Pohlmann AR (2012). Hemocompatibility of poly(ɛ-caprolactone) lipid-core nanocapsules stabilized with polysorbate 80-lecithin and uncoated or coated with chitosan. Int J Pharm.

[54] Bennett MR, Sinha S, Owens GK (2016). Vascular smooth muscle cells in atherosclerosis. Circ Res.

[55] Gimbrone MJ, Garcia-Cardena G (2016). Endothelial cell dysfunction and the pathobiology of atherosclerosis. Circ Res.

[56] Tabas I, Garcia-Cardena G, Owens GK (2015). Recent insights into the cellular biology of atherosclerosis. J Cell Biol.

[57] Oishi Y, Manabe I (2018). Macrophages in inflammation, repair and regeneration. Int Immunol.

[58] Lobatto ME, Calcagno C, Millon A, Senders ML, Fay F, Robson PM (2015). Atherosclerotic plaque targeting mechanism of long-circulating nanoparticles established by multimodal imaging. ACS Nano.

[59] Kim Y, Lobatto ME, Kawahara T, Lee Chung B, Mieszawska AJ, Sanchez-Gaytan BL (2014). Probing nanoparticle translocation across the permeable endothelium in experimental atherosclerosis. Proc Natl Acad Sci U S A.

[60] Jiang Q, Liu Y, Guo R, Yao X, Sung S, Pang Z (2019). Erythrocyte-cancer hybrid membrane-camouflaged melanin nanoparticles for enhancing photothermal therapy efficacy in tumors. Biomaterials.

