# Supplementary material for: Macrophage membrane functionalized biomimetic nanoparticles for targeted anti-atherosclerosis applications
Source: Theranostics. 2021 Jan 1;11(1):164–80. doi: 10.7150/thno.47841 (PMC7681077; doi:10.7150/thno.47841)
Supplement: Supplementary file 1 — Supplementary figures and tables. [file thnov11p0164s1.pdf]

Supplementary materials

Macrophage membrane functionalized biomimetic nanoparticles for targeted anti-atherosclerosis applications

Yi Wang[1,2‡], Kang Zhang[1‡], Tianhan Li[1], Ali Maruf[1], Xian Qin[1], Li Luo[1], Yuan Zhong[1], Juhui Qiu[1], Sean McGinty[3], Giuseppe Pontrelli[4], Xiaoling Liao[2*], Wei Wu[1*], Guixue Wang[1*]

[1]Key Laboratory for Biorheological Science and Technology of Ministry of Education, State and Local Joint Engineering Laboratory for Vascular Implants, Bioengineering College of Chongqing University, Chongqing, 400030, China

[2]Chongqing Key Laboratory of Nano/Micro Composite Material and Device, School of Metallurgy and Materials Engineering, Chongqing University of Science and Technology, Chongqing, 401331, China

[3]Division of Biomedical Engineering, University of Glasgow, UK

[4]Istituto per le Applicazioni del Calcolo - CNR, Via dei Taurini 19, 00185, Roma, Italy

[‡]These authors contributed equally to this work.

*Corresponding authors: wanggx@cqu.edu.cn (Guixue Wang); david2015@cqu.edu.cn (Wei Wu); 2007099@cqust.edu.cn (Xiaoling Liao)

Table S1. Loading and encapsulation efficiency of RAPNPs.

	μg/mg	Drug Loading Efficiency (%)	Drug Encapsulating Efficiency (%)
RAPNPs	68.4 ± 0.07	6.87	76.3

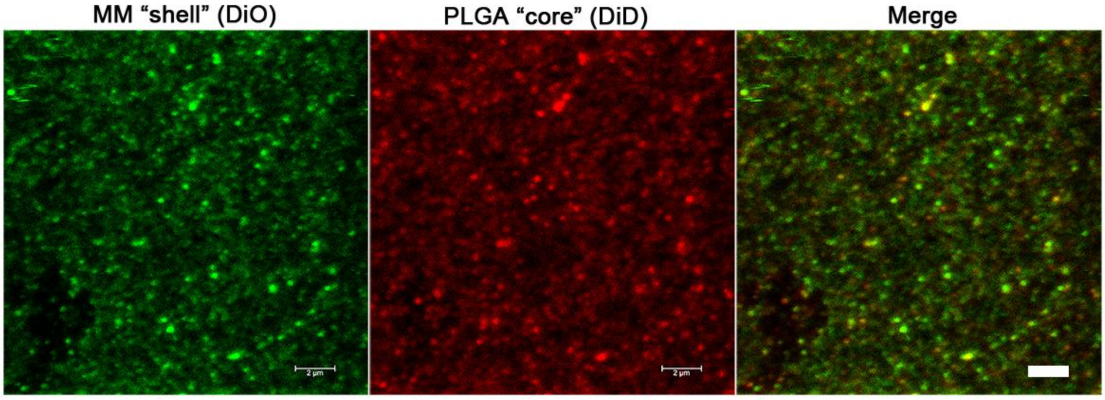

Figure S1. Representative fluorescence images of MM coated nanoparticles, the MM was stained by DiO and the PLGA NPs labeled by DiD (Scale bars, 2 μm).

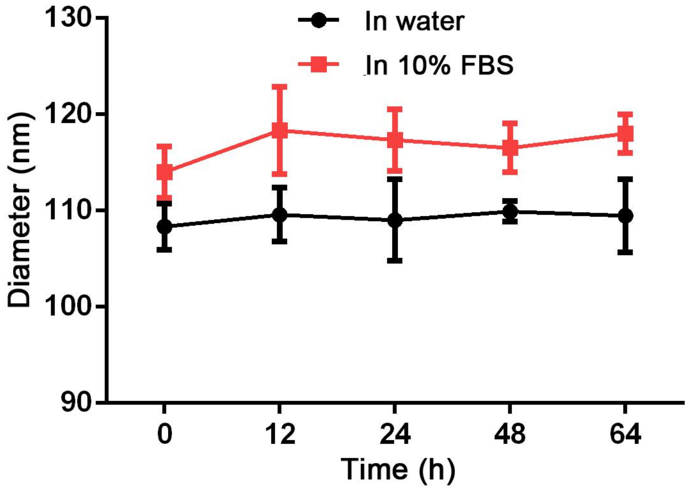

Figure S2. The size change tendency of MM/RAPNPs in water and medium containing 10% FBS at room temperature (n = 3, mean ± SD).

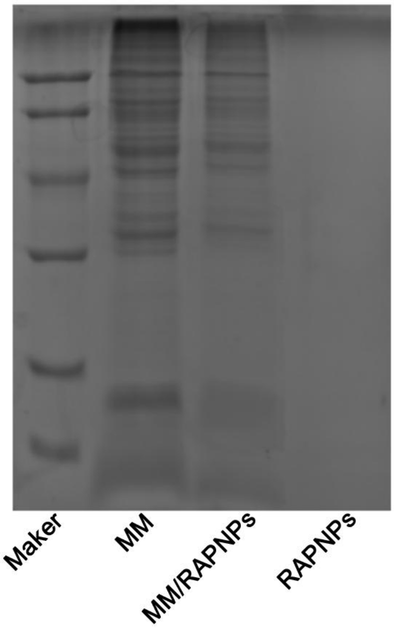

Figure S3. The protein profiles of the MM, MM/RAPNPs and RAPNPs determined by SDS-PAGE electrophoresis assay.

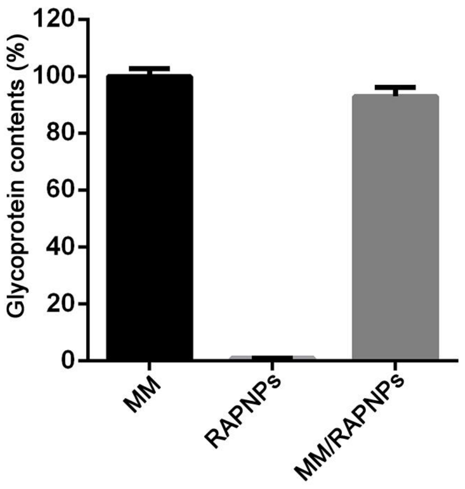

Figure S4. Quantification of the relative glycoprotein content (n = 3, mean ± SD).

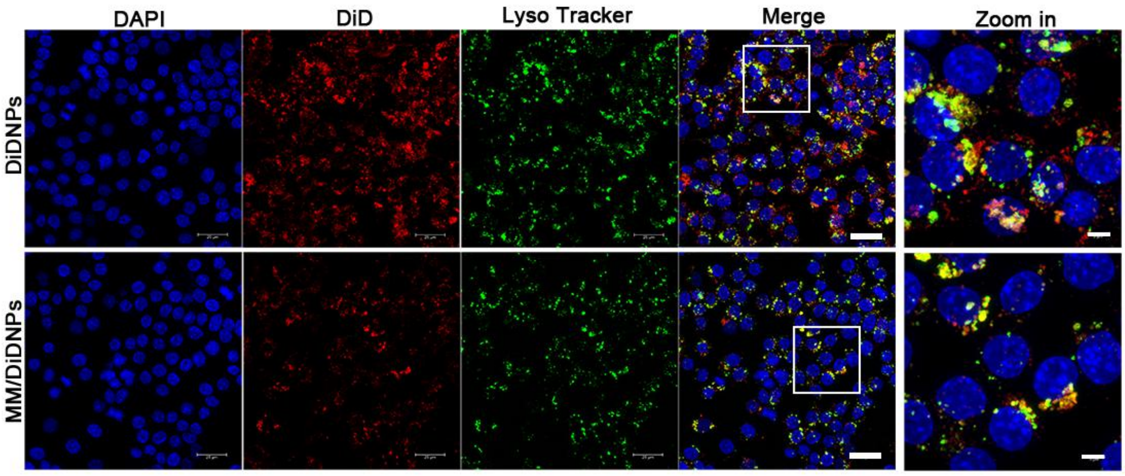

Figure S5. Fluorescence images of DiDNPs and MM/DiDNPs internalized by RAW264.7 cells after 4 h of incubation. Lysosomes were stained with LysoTracker (green), while nuclei were stained with DAPI (blue). Scale bars, 25 μm (left) and 5 μm (right).

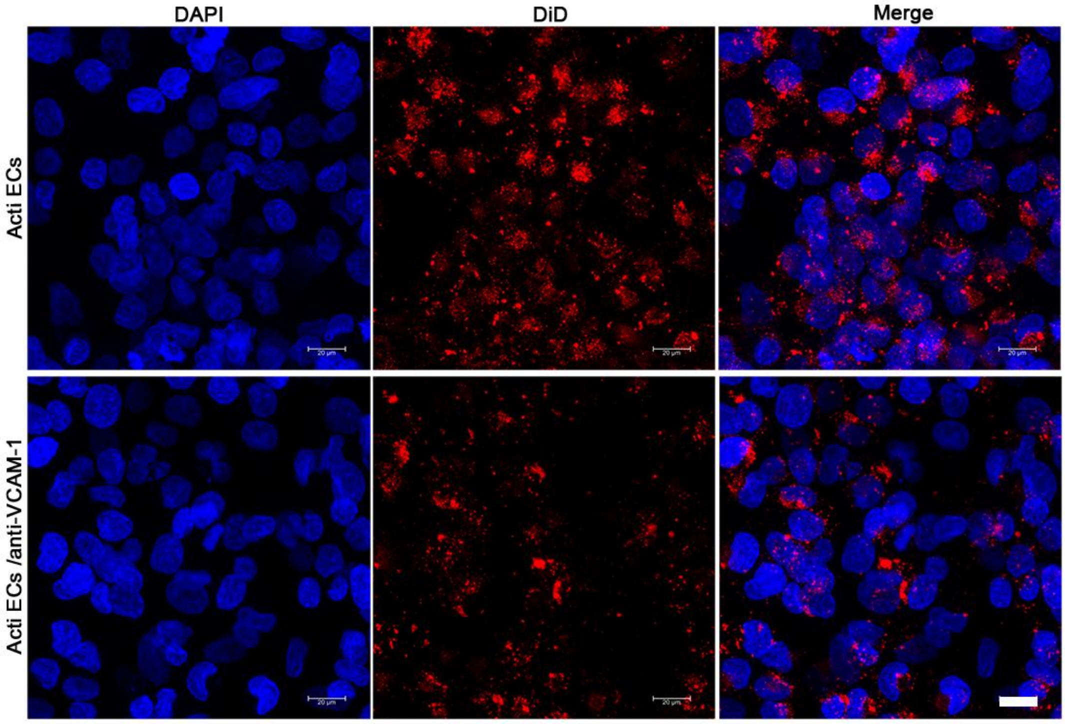

Figure S6. Fluorescence images of MM/DiDNPs internalized by activated HUVECs or VCAM-1 antibodies blocked activated HUVECs (scale bar =20 μm).

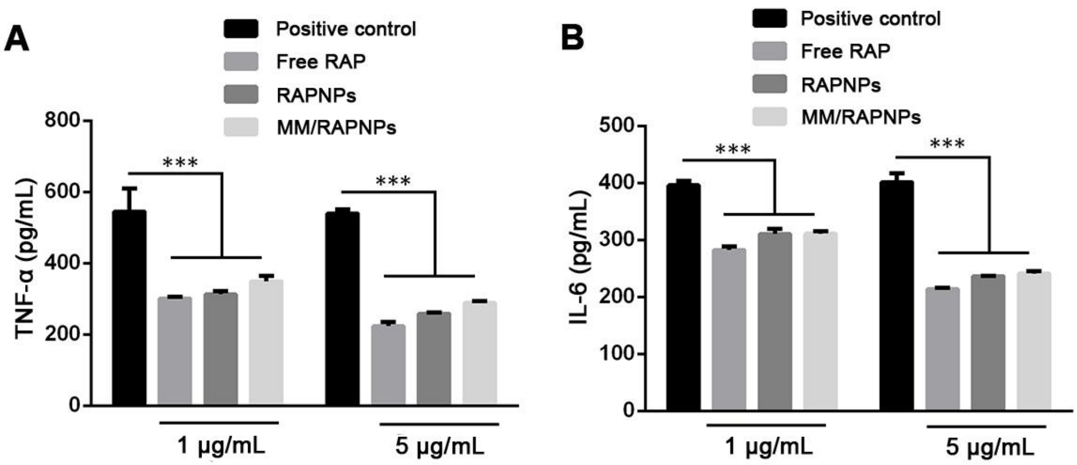

Figure S7. Typical inflammatory cytokines TNF-α (A) and IL-6 (B) secreted by RAW264.7 macrophages after different treatments for 24 h (n=3, mean ± SD). ***p < 0.001.

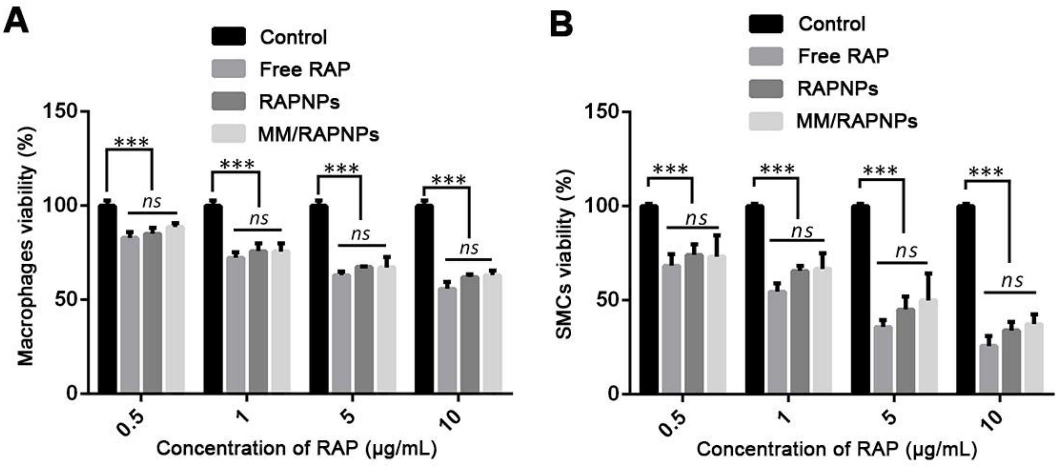

Figure S8. The anti-proliferation activities of RAP and RAP loaded NPs in RAW 264.7 cells (A) and SMCs (B) (*n*=5, mean ± SD). ***$p < 0.001$. *ns*, no significance.

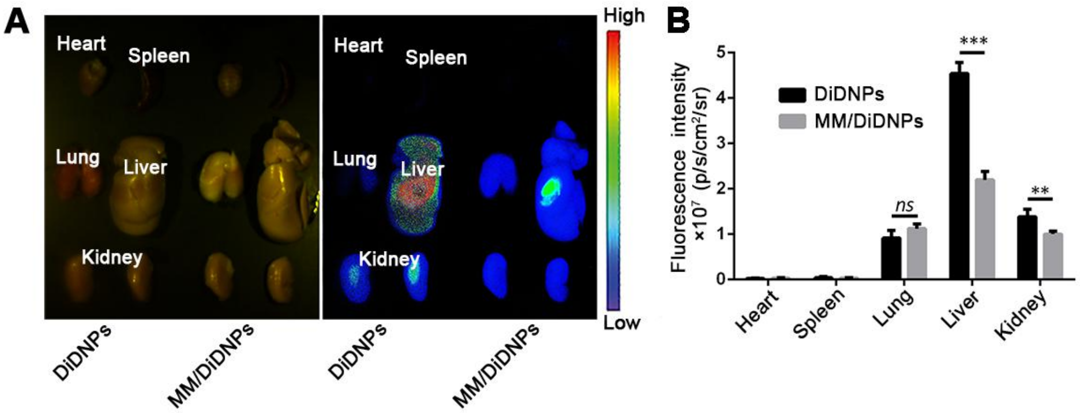

Figure S9. (A) *Ex vivo* fluorescence images of the heart, spleen, lung, liver, kidney at 24 h post-injection. (B) Quantitative analysis of fluorescent signals from main organs at 24 h post-injection (n = 3, mean ± SD). (**p < 0.01 and ***p < 0.001, *ns*, no significance).

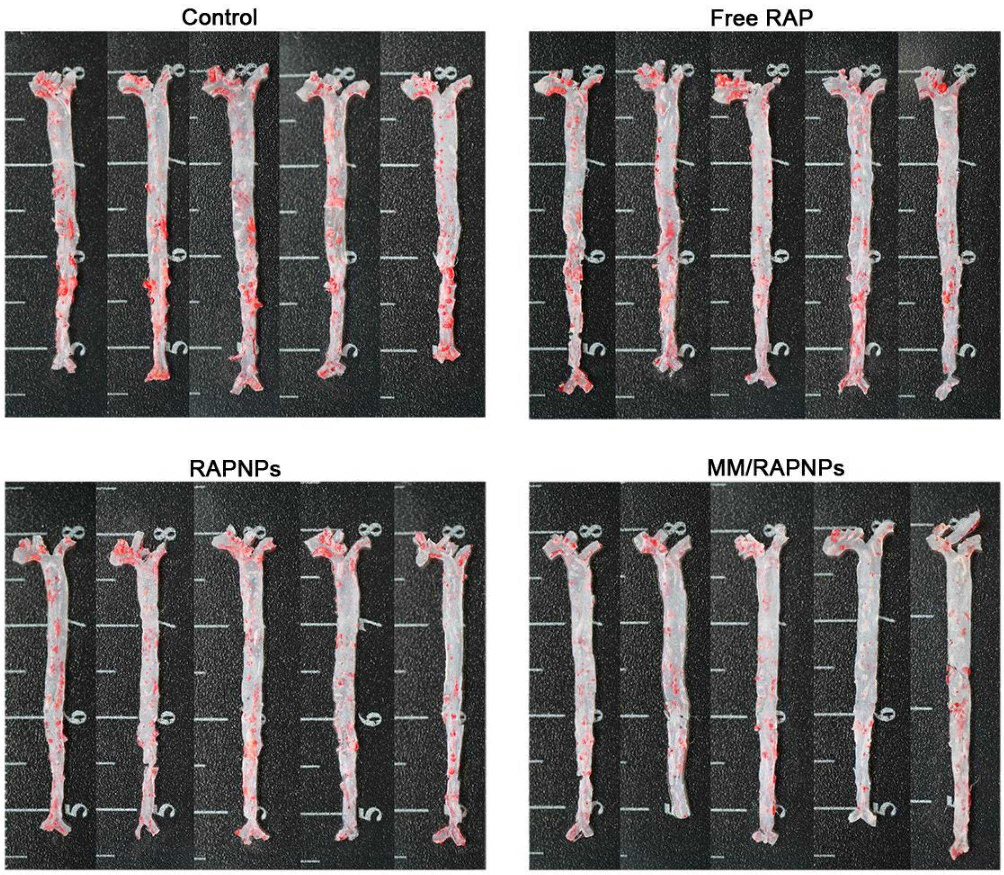

Figure S10. Images of ORO-stained en face aortic preparations in different treatment groups.

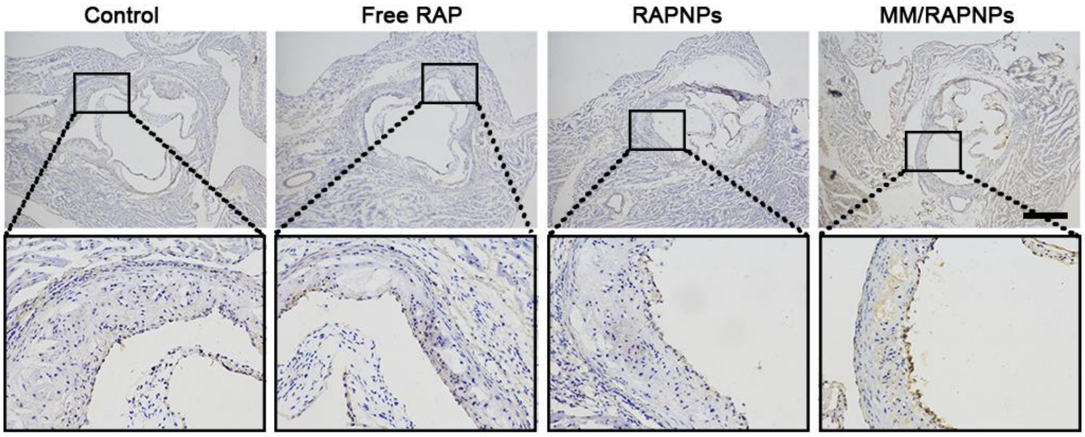

Figure S11. Immunohistochemistry analysis on the sections of aortic roots from ApoE$^{-/-}$ mice post different treatments. Representative photographs of immunohistochemistry staining with antibodies to CD31 (scale bar = 500 μm).

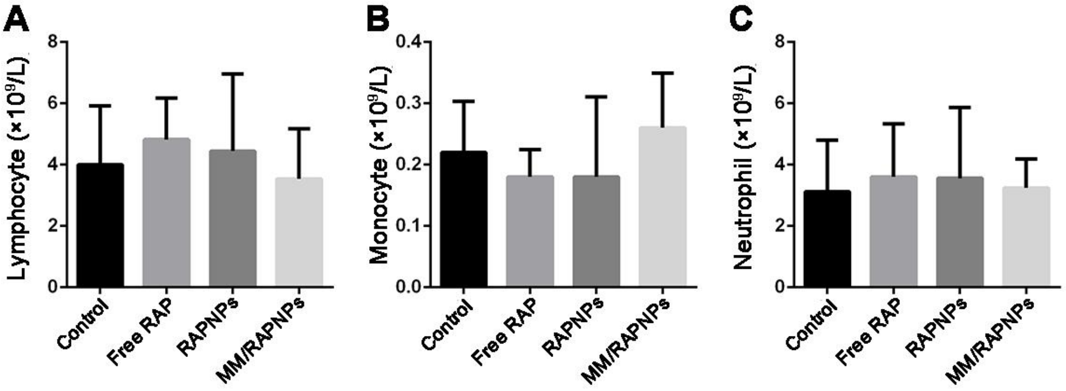

Figure S12. Blood cell counts of immune-associated cells including lymphocyte, monocyte and neutrophil after one month treatment (*n*=5).

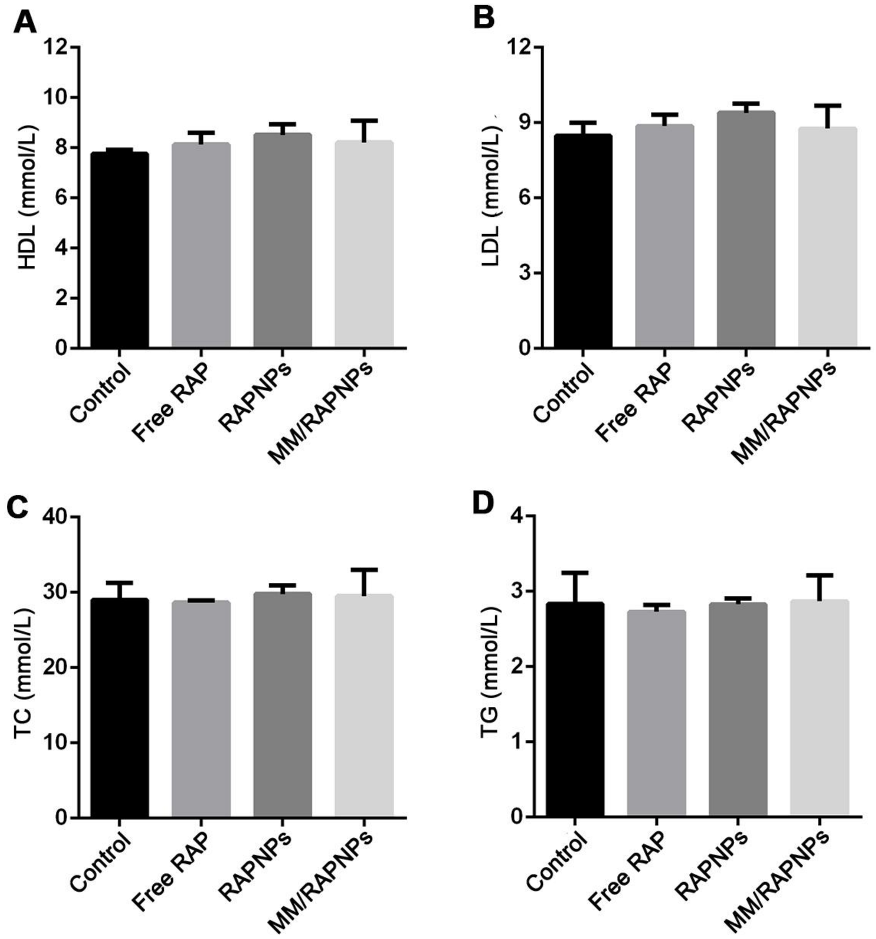

Figure S13. Results of blood lipid test (A-D). HDL, high density lipoprotein cholesterol; LDL, low density lipoprotein cholesterol; TC, the serum total cholesterol; TG, triglyceride; (*n* = 5, mean ± SD).
